# Klotho exerts protection in chronic kidney disease associated with regulating inflammatory response and lipid metabolism

**DOI:** 10.1186/s13578-024-01226-4

**Published:** 2024-04-07

**Authors:** Junhui Liu, Huaicheng Wang, Qinyu Liu, Shushu Long, Yanfang Wu, Nengying Wang, Wei Lin, Gang Chen, Miao Lin, Junping Wen

**Affiliations:** 1 Shengli Clinical Medical College of Fujian Medical University, Fujian Medical University, Fuzhou, China; 2 Department of Endocrinology, Fujian Provincial Hospital, Shengli Clinical Medical College of Fujian Medical University, Fuzhou, China; 3 Department of Nephrology, Provincial Clinical College, Fujian Provincial Hospital, Fujian Medical University, Fuzhou, China

**Keywords:** Klotho, Chronic kidney disease, Inflammatory response, Lipid metabolism, Biomarker

## Abstract

**Background:**

The anti-aging protein Klotho plays a protective role in kidney disease, but its potential as a biomarker for chronic kidney disease (CKD) is controversial. Additionally, the main pathways through which Klotho exerts its effects on CKD remain unclear. Therefore, we used bioinformatics and clinical data analysis to determine its role in CKD.

**Results:**

We analyzed the transcriptomic and clinical data from the Nephroseq v5 database and found that the *Klotho* gene was mainly expressed in the tubulointerstitium, and its expression was significantly positively correlated with estimated glomerular filtration rate (eGFR) and negatively correlated with blood urea nitrogen (BUN) in CKD. We further found that *Klotho* gene expression was mainly negatively associated with inflammatory response and positively associated with lipid metabolism in CKD tubulointerstitium by analyzing two large sample-size CKD tubulointerstitial transcriptome datasets. By analyzing 10-year clinical data from the National Health and Nutrition Examination Survey (NHANES) 2007–2016, we also found that Klotho negatively correlated with inflammatory biomarkers and triglyceride and positively correlated with eGFR in the CKD population. Mediation analysis showed that Klotho could improve renal function in the general population by modulating the inflammatory response and lipid metabolism, while in the CKD population, it primarily manifested by mediating the inflammatory response. Restricted cubic spline (RCS) analysis showed that the optimal concentration range for Klotho to exert its biological function was around 1000 pg/ml. Kaplan–Meier curves showed that lower cumulative hazards of all-cause mortality in participants with higher levels of Klotho. We also demonstrated that Klotho could reduce cellular inflammatory response and improve cellular lipid metabolism by establishing an in vitro model similar to CKD.

**Conclusions:**

Our results suggest that Klotho exerts protection in CKD, which may be mainly related to the regulation of inflammatory response and lipid metabolism, and it can serve as a potential biomarker for CKD.

**Supplementary Information:**

The online version contains supplementary material available at 10.1186/s13578-024-01226-4.

## Background

*αKlotho* was initially identified as a gene associated with anti-aging effects, as its deficiency resulted in a syndrome similar to premature aging [1]. The Klotho family currently consists of *αKlotho*, *βKlotho* and *γKlotho* [2]. In this study, Klotho specifically refers to *αKlotho*. Human Klotho is expressed in various tissues including neuronal, reproductive, arterial and endocrine tissues [3]. However, it is predominantly found in the distal tubules of the kidney and parathyroid glands [4, 5]. Human Klotho is a transmembrane protein with a molecular weight of 130 kDa and consists of 1012 amino acid residues [6]. The extracellular structural domain of the membrane Klotho consists of two tandem repeat sequences known as Kl1 and Kl2. These sequences can be cleaved by the metalloproteases ADAM-10 and ADAM-17, releasing soluble Klotho into the circulation [7]. The molecular weight of soluble Klotho is about 65 kDa [8, 9]. As a circulating substance, Klotho exerts multiple biological effects on various organs [10].

Under normal physiological conditions, soluble Klotho primarily originates from the kidneys [11]. It offers protection against various systemic diseases such as type 2 diabetes [12], interstitial lung disease [13], nonalcoholic fatty liver disease [14], and cardiovascular disease [15]. In particular, Klotho can enhance the affinity of the fibroblast growth factor receptor (FGFR) and act as a co-receptor for fibroblast growth factor 23 (FGF23), which regulates the stability of phosphorus and vitamin D metabolism in the body [16]. Additionally, Klotho plays an essential protective role in kidney disease. The expression of Klotho is reduced in cases of acute kidney injury. At the same time, supplementation with artificial recombinant Klotho has shown promise in improving acute kidney injury caused by contrast agents [17], ischemia–reperfusion injury [18], and sepsis [19]. Furthermore, recombinant Klotho (rKlotho) has the potential to prevent the progression of acute kidney disease to CKD [20]. In patients with CKD, Klotho expression is also reduced [21]. The decrease in serum Klotho levels begins in late CKD stage 2 [22], while urinary Klotho levels decrease significantly in early CKD stage 1 and continue to decline as CKD progresses [23]. Additionally, the reduction in human urinary Klotho is directly correlated with a decrease in eGFR [24]. Urinary Klotho has a more sensitive correlation with renal function, indirectly suggesting that Klotho is primarily of renal origin. In an animal model of CKD, Klotho-deficient mice exhibited more severe kidney damage, developed CKD more rapidly, and increased fibrosis compared to wild-type mice. In contrast, Klotho over-expressing mice showed less renal dysfunction and fibrosis, providing a protective mechanism through upregulation of autophagy [25]. These findings suggest a close association between Klotho deficiency and the development as well as progression of CKD.

However, the use of Klotho as a biomarker for CKD remains controversial. Several prospective cohort and cross-sectional studies have produced different results [26–29]. One study has specifically analyzed the reasons for these discrepancies and advocated for improvement of the experimental detection performance of soluble Klotho [30]. Recent meta-analyses have suggested that Klotho could be an early biological marker for CKD [31–33]. However, the reliability of this conclusion is limited by the small sample sizes used in these analyses. In particular, there is a lack of evidence from the combined genetic and clinical data analysis that could increase the reliability of research findings. Additionally, Klotho is known to exert protective effects in kidney disease through various regulatory pathways, including anti-apoptosis, anti-senescence, anti-oxidative stress, anti-fibrosis, anti-inflammatory effects, induction of autophagy, promotion of angiogenesis and maintenance of mineral metabolism stability [34]. Nonetheless, the primary pathways through which Klotho regulates renal function in CKD remain unclear.

Therefore, our study aims to provide reliable evidence from the combined analysis of genetic and clinical data to support Klotho as a potential biomarker for CKD. In addition, we strive to identify the primary pathways through which Klotho exerts its role in CKD by integrating bioinformatics analysis, clinical data analysis and in vitro experiments.

## Methods

### Analysis of *Klotho* gene expression in kidney and its relationship with renal function in CKD

*KL* (*Kloth*o gene) expression data in major organs/tissues throughout the body were obtained from GTEx (Genotype-Tissue Expression) RNA-seq data in the Human Protein Atlas (www.proteinatlas.org). *KL* expression in Glomerulus and tubulointerstitium was studied using data (GSE21785) from the gene expression study published by Lindenmeyer et al [35]. *KL* expression in different renal cell types was determined in a publicly available human adult kidney single nucleus RNA sequencing (snRNA-seq) data from GSE118184. The adult kidney snRNA-seq matrix data was provided by Wu H et al [36]. Using the R language ‘‘Seurat’’ package (version 4.3) for cellular clustering analysis, we identified the main 12 cell clusters and labeled them with different kidney cell type marker genes and visualized them with a tSNE plot. The *KL* expression in each cell cluster was demonstrated using the FeaturePlot, VlnPlot, and DotPlot functions in the "Seurat" package. We extracted *KL* expression data from the Ju CKD Glom and Ju CKD TubInt datasets in the Nephroseq v5 database (http://v5.nephroseq.org/) [37, 38], and we also extracted renal function data corresponding to CKD patients, including eGFR and BUN. According to non-normal distribution data, the Mann–Whitney test was used to compare the expression of *KL* in CKD glomeruli and normal glomeruli, and we also compared *KL* expression in CKD tubulointerstitium and normal tubulointerstitium. The correlation of *KL* expression in glomeruli and tubulointerstitium with eGFR and BUN in CKD was analyzed using Spearman correlation. We also extracted *KL* expression and eGFR data from the Sampson Nephrotic Syndrome TubInt and Woroniecka Diabetes TubInt datasets in the Nephroseq v5 database [39, 40]. We then analyzed the correlation between *KL* expression and eGFR in the two independent data using Spearman correlation.

### GEO data download and pre-processing

Two large sample sizes of CKD tubulointerstitial transcriptome datasets, GSE104954 and GSE108112, were downloaded from the GEO database (https://www.ncbi.nlm.nih.gov/geo/). Samples for the GSE104954 dataset were RNA extracted from tubulointerstitium and hybridized on Affymetrix Human Genome U133A arrays or Affymetrix Human Genome U133 Plus 2.0 arrays. The annotation of these samples was performed using the Human Entrez Gene ID custom CDF version 19. Since the GSE104954 dataset was processed using two batches of data, we used the script and the ‘‘SVA’’ package in the R software to perform the batch correction [41]. 190 samples from GSE104954 were used for immune cell infiltration analysis, comprising 32 cases of lupus nephritis (LN), 25 cases of IgA nephropathy (IgAN), 21 cases of rapidly progressive glomerulonephritis (RPGN), 20 cases of hypertensive nephropathy (HT), 18 cases of membranous glomerulonephritis (MGN), 17 cases of diabetic nephropathy (DN), 13 cases of focal segmental glomerulosclerosis (FSGS), 13 cases of minimal change disease (MCD), 6 cases of thin basement membrane nephropathy (TBMN), 4 cases of focal segmental glomerulosclerosis and minimal change disease (FSGS-MCD) and 21 cases of living donors (LD). The remaining 169 samples after excluding 21 LD samples were formed as the GSE104954 (CKD) dataset. Samples from the GSE108112 dataset were RNA extracted from tubulointerstitium and hybridized on Affymetrix Human Gene 2.1 ST arrays. The annotation of these samples was performed using the Human Entrez Gene ID custom CDF version 19. 169 samples from the GSE108112 dataset were used for immune cell infiltration analysis, including 57 cases of ANCA-associated vasculitis (AAV), 46 cases of focal segmental glomerulosclerosis (FSGS), 43 cases of membranous glomerulonephritis (MGN), 18 cases of minimal change disease (MCD) and 5 cases of living donors (LD). The remaining 164 samples after excluding 5 LD samples were formed as the GSE108112 (CKD) dataset.

### *Klotho* gene functional enrichment analysis

We screened the genes significantly associated with *KL* expression by Spearman correlation analysis (with correlation coefficient R < − 0.5 or R > 0.5, P < 0.05) in the GSE104954 (CKD) dataset and the GSE108112 (CKD) dataset, then uploaded to the Database for Annotation, Visualization, and Integrated Discovery (DAVID, v2023q1, https://david.ncifcrf.gov/tools.jsp). The official gene symbol was selected as the identifier, and Homo sapiens was selected as the species. Lastly, the enrichment results were obtained from Gene Ontology (GO) analysis and Kyoto Encyclopedia of Genes and Genomes (KEGG) pathway analysis. This study shows the top five enrichment results in ascending order of P-value (P < 0.05).

### Gene set variation analysis (GSVA)

The gene lists associated with inflammatory response and the gene lists associated with lipid metabolism were obtained from the AmiGO 2 portal website (http://amigo.geneontology.org/amigo/landing). The functional enrichment score of inflammatory response and lipid metabolism for each CKD tubulointerstitium sample was calculated using the R language "GSVA" package (version 1.46.0) under default parameters [42]. Heatmaps of the enrichment results were drawn with the R language "pheatmap" package (version 1.0.12). The correlation between *Klotho* gene expression and inflammatory response as well as lipid metabolism was determined by Spearman correlation analysis.

### Immune cell infiltration analysis

We used the online tool CIBERSORTx (https://cibersortx.stanford.edu/) to calculate the relative abundance of 22 immune cells in the GSE104954 and GSE108112 samples. The percentage of 22 immune cells in the CKD and Control groups were compared separately in GSE104954. Since the percentage of Eosinophils was zero in GSE104954 (CKD), and the percentage of Mast cells activated also was zero in GSE108112 (CKD), we only analyzed 21 immune cells’ correlation with *Klotho* gene expression by Spearman correlation analysis.

### Cell lines culture and treatment

HK-2 (Procell, China) is a cell line that originates from immortalized human proximal tubule epithelial cells. HK-2 cells were cultured in a 37 °C humidified atmosphere with 5% CO2. The medium was DMEM/Ham’s F-12 which was supplemented with 10% fetal bovine serum (FBS), 100 U/ml penicillin, and 100 ug/ml streptomycin. At 60% ~ 80% confluence, cells were stimulated with TGF-β1 and palmitic acid (TPA) at a final concentration of 5 ng/mL TGF-β1 and 200 umol/L palmitic acid for 48 h with and without recombinant human Klotho (rKlotho, 100 ng/ml) [43–46]. After 48 h, the cell culture medium was collected separately, centrifuged at 2500 rpm for 10 min to remove cell debris, filtered through a 0.22 μm filter and stored in clean test tubes. The collected medium was used for the subsequent experiments.

THP-1 (Procell, China) is a cell line that originates from immortalized human monocyte-like cells. THP-1 cells were cultured in RPMI1640 medium supplemented with 10% FBS, 100 U/ml penicillin, and 100 ug/ml streptomycin. At 60% ~ 80% confluence, cells were used for transwell invasion assay.

### Transwell invasion assay

100 ul of diluted GelNest matrix (NEST, China) was spread evenly on the upper chamber of the transwell device (NEST, China) and incubated at 37 °C in a culture incubator for 1 h to promote solidification. THP-1 cells (5 × 10^4^ cells/well) were added to the upper chamber with 200 μl of FBS-free RPMI1640 medium plus or without plus rKlotho (100 ng/ml). FBS-free RPMI1640 medium and 10% FBS RPMI1640 medium or conditioned medium obtained from HK-2 with normal treatment, TPA treatment or TPA + rKlotho treatment for 48 h were added to the lower chamber of the transwell culture system in a volume of 600 μl. After incubation for 24 h, THP-1 cells at the bottom of chamber membrane were fixed with 4% paraformaldehyde for 10 min and stained with 0.1% crystal violet for 10 min. The cells inside the upper chamber were gently removed with a wet cotton swab, and the chamber membrane was imaged. The number of cells in five randomly selected fields of view was counted.

### Western blotting

The total protein of HK-2 was extracted with RIPA buffer (Beyotime, China) according to the manufacturer's instructions. Protein samples were subjected to 10% SDS-PAGE and then transferred to polyvinylidene fluoride (PVDF) membranes (Millipore, USA). After blocking with 5% BSA (Solarbio, China), the membranes were incubated with corresponding primary antibodies: anti-Klotho (1:2000, Proteintech, USA), anti-pSmad3 (1:1000, ABclonal, China), anti-Smad3 (1:2000, Proteintech, USA), anti-PPARα (1:1000, Proteintech, USA), Anti-PGC1α (1:1000, ABclonal, China) and anti-GAPDH (1:10,000, Proteintech, USA). After incubation with appropriate secondary antibodies, the western blots were visualized using the ECL Western Blotting Substrate (Meilunbio, China). The signals were quantified with ImageJ software.

### Real-time PCR

The total RNA of HK-2 was extracted with RNAiso Plus (TaKaRa, China) according to the manufacturer's instructions. Reverse transcription was carried out with a PrimeScript™ RT reagent Kit (TaKaRa, China). The specific sequence of each human primer pair was shown in Additional file 1: Table S1. Real-time PCR analysis was performed using the LightCycler 480 II System (Roche, Switzerland) with TB Green® Premix Ex Taq^™^ II (TaKaRa, China). The samples were subjected to PCR analysis using the following cycling parameters: 95 °C for 30 s, then 95 °C for 5 s and 60 °C for 30 s for 40 cycles, 95 °C for 5 s, 60 °C for 60 s, 95 °C for 15 s. Relative gene expression levels were normalized to *GAPDH* gene expression.

### Enzyme-linked immunosorbent assay (ELISA)

The concentrations of secreted TNF-α, IL-1β, and IL-6 in the culture supernatants of HK-2 were determined using their respective ELISA kits (MultiSciences, China) according to the manufacturer's instructions.

### Oil red O staining

HK-2 cells were inoculated in 12-well plates at 1 × 10^**5**^ cells per well. We treated cells with TPA for 48 h with or without rklotho. At the end of treatment, cells were fixed with 4% paraformaldehyde for 30 min and immersed with 60% isopropanol for 30 s. Then, cells were stained with Oil Red O (Solarbio, China) for 20 min at room temperature. After washing with distilled water, the images were obtained under an inverted microscope (Leica, Germany) at 200×magnification. The lipid droplets were quantified with ImageJ software.

### Mitochondrial visualization

According to the manufacturer's instructions, mitochondria were visualized with MitoTracker (Beyotime, China). Fifteen fields of view were randomly selected from each sample to examine the morphology of mitochondria. Semi-quantitative analysis was performed by calculating the percentage of cells with altered mitochondrial pattern (in terms of fragmentation and perinuclear redistribution) out of the total cells in each field of view [44]. Analysis of mean branch length per mitochondrion was performed using the ImageJ software.

### Triglyceride measurement

HK-2 cells were inoculated in 6-well plates at 5 × 10^**5**^ cells per well. We treated cells with TPA for 48 h with or without rklotho. At the end of treatment, cells were broken using Scientz-IID Ultrasonic Cell Crusher (Scientz, China), and the cell supernatants were collected for triglyceride measurement. Triglyceride concentrations in cell supernatants were determined using a triglyceride (TG) colorimetric assay kit (Elabscience, China) according to the manufacturer's instructions.

### NHANES clinical data and participants

The data used in this study were obtained from the NHANES in the United States. NHANES is a nationally representative observational survey conducted by the National Center for Health Statistics (NCHS). Its purpose is to assess the health and nutrition status of the non-institutionalized population in the country. The survey is conducted biennially and collects various information such as demographics, physical status, laboratory tests, dietary patterns and medical history. Prior to conducting the survey, the Research Ethics Review Committee of NCHS approved it and ensured that all participants provided informed consent. For more detailed statistical data, please refer to the NHANES portal (https://www.cdc.gov/nchs/nhanes/).

For this study, a total of 50,588 participants (aged 40 to 79 years) in the NHANES cycles between 2007 and 2016 were included. Relevant information was essential to investigate the association of Klotho with inflammatory biomarkers, lipid biomarkers, renal function biomarkers and mortality. Hence, the following participants were excluded: (1) participants with missing serum Klotho data; (2) participants without survival information; (3) pregnant women; (4) participants taking lipid-lowering medication; (5) participants without complete covariates. The participant selection flowchart is shown in Fig. 7. 9719 participants (including 1520 CKD) were enrolled in the survival analysis. 9680 participants (including 1514 CKD) had complete inflammatory biomarkers, 9713 participants (including 1519 CKD) had complete renal function biomarkers, 4602 participants (including 685 CKD) had data of LDL, and 9711 participants (including 1520 CKD) had complete other lipid biomarkers were enrolled in the association analysis, respectively. 9675 participants (including 1514 CKD) with complete data of renal function and inflammation biomarkers, 4601 participants (including 685 CKD) with complete data of renal function and LDL, and 9709 participants (including 1519 CKD) with complete data of renal function and other lipid biomarkers were enrolled in the mediation analysis, respectively. CKD is characterized by abnormalities in kidney structure or function that persist for more than 3 months and have an impact on health. Its diagnostic criteria are outlined in the guideline of Kidney Disease Improving Global Outcomes (KDIGO) [47].

### Measurement of major serum biomarkers

Inflammatory biomarkers include white blood cell (WBC), neutrophil (Neu), lymphocyte (Lym), monocyte (Mono), systemic immune-inflammation index (SII), pan-immune-inflammation value (PIV), systemic inflammatory response index (SIRI) and systemic chronic inflammation (SCI) which includes neutrophil-to-lymphocyte ratio (NLR), Monocyte-to-lymphocyte ratio (MLR) and platelet-to-lymphocyte ratio (PLR). Complete blood cell counts on blood specimens were analyzed on the Beckman UniCel® DxH 800 Analyzer. The following equations were used to calculate SII, PIV, SIRI, NLR, MLR, and PLR according to previous studies [48–51]. SII = (platelet count × neutrophil count) / lymphocyte count; PIV = (neutrophil count × platelet count × monocyte count)/Lymphocyte count; SIRI = (neutrophil count × monocyte count)/lymphocyte count; NLR = neutrophil count/lymphocyte count; MLR = monocyte count/lymphocyte count; PLR = platelet count/lymphocyte count. Lipid biomarkers include high density lipoprotein (HDL), low density lipoprotein (LDL), total cholesterol (TC) and triglyceride (TG). HDL, LDL and TC serum concentrations were analyzed by Cobas 6000 Chemistry Analyzer; TG serum concentration was analyzed by Beckman UniCel® DxC 800 / DxC 660i Synchron Access Clinical Systems. Renal function biomarkers include eGFR, serum creatinine, serum urea nitrogen, uric acid and Urine Albumin-to-Creatinine Ratio (UACR). Serum creatinine, serum urea nitrogen and uric acid were analyzed by Beckman UniCel® DxC 800 / DxC 660i Synchron Access Clinical Systems. The eGFR was calculated using the Chronic Kidney Disease Epidemiology Collaboration (CKD-EPI) equation [52]. UACR was calculated using the equation: UACR (mg/g) = urinary albumin (mg/dL) / urinary creatinine (g/dL). Soluble Klotho was tested using the commercially available ELISA kit produced by IBL International, Japan. Serum samples were analyzed twice and the average of the two values was used to calculate the final values. In each ELISA plate, two quality control samples of low and high Klotho concentrations were also analyzed in duplicate. The analysis results were automatically transferred from the instrument to the laboratory Oracle Management System and the results were evaluated by the area supervisor. Samples with duplicate results exceeding 10% were flagged as requiring duplicate analysis. If the value of one quality control sample was not within 2SD of the assigned value, the entire analysis run was rejected and the sample was re-analyzed. The detection sensitivity was 6 pg/mL and the final values for all samples exceeded this limit. Above all details on laboratory methods and protocols are provided in the NHANES Laboratory Procedure Manual.

### Covariates

Population demographic information comprised gender (male and female), age, race/ethnicity (Mexican American, Non-Hispanic White, Non-Hispanic Black, and other races), and educational level (< 11th grade, High school graduate, Some college or AA degree and College graduate or above). The examination variable was body mass index (categorized, ≤ 25 as normal weight, 25–30 as overweight, > 30 as obese), calculated as weight divided by the square of height. Questionnaire variables comprised smoking status (categorized, less than 100 times in lifetime as never smoked, 100 or more times but not now smoking as former, 100 or more times now smoking as current). Hypertension was defined as individuals who met at least one of the following criteria: average systolic/diastolic blood pressure ≥ 140/90 mmHg, self-reported hypertension, or currently using antihypertensive medication. Diabetes mellitus (DM) was defined as individuals who met at least one of the following criteria: the doctor told them they had diabetes, fasting glucose (mmol/l) ≥ 7.0, glycohemoglobin HbA1c (%) > 6.5, random blood glucose (mmol/l) ≥ 11.1, two-hour OGTT blood glucose (mmol/l) ≥ 11.1, or use of insulin or diabetes medication. Cardiovascular disease (CVD) was confirmed by asking participants if they had been told by the physician that they had at least one of the following medical conditions: heart attack, coronary heart disease, angina and congestive heart failure.

### Statistics analysis and methods

All analyses were conducted using R software (version 4.2.3) and GraphPad Prism software (version 8.0.1). snRNA-seq matrix data analysis was performed using the "Seurat" package (version 4.3). Mann–Whitney test was used to compare continuous non-normally distributed data between groups, and Spearman correlation analysis was used for continuous non-normally distributed data. The heatmap was generated using the ‘‘pheatmap’’ package. In the in vitro experiments, the differences between groups were analyzed using one-way ANOVA or unpaired t-tests. The NHANES clinical data description and statistical analytics in this study were performed using the ‘‘Survey’’ package in the R software, employing complex weighted methods. Baseline characteristics were compared between non-CKD and CKD groups using a t-test for continuous variables and the χ2 statistical method for categorical variables. Continuous variables were described as mean ± standard deviation (SD), while categorical variables were presented as n (%). Klotho levels were divided into tertiles, with the lowest tertile being the reference group. The generalized linear model (GLM) was used to evaluate the relationships between serum Klotho levels and inflammatory biomarkers, lipid biomarkers and renal function biomarkers. To further investigate the dose–response curves between Klotho levels and inflammatory biomarkers, lipid biomarkers, and renal function biomarkers, restricted cubic splines (RCS) with 3 knots at the 10th, 50th, and 90th percentiles of the Klotho level distribution were used. To determine whether Klotho can improve renal function by mediating inflammatory response or lipid metabolism, a causal mediation analysis was conducted using the ‘‘mediation’’ R package to estimate the indirect effect, direct effect and total effect. It is expected that the mediators would have associations with both the exposure and outcome variables. The mediation analysis was performed with adjustment for the same covariates used in the adjusted model. The differences in cumulative hazards of all-cause mortality among tertiles of Klotho were compared through Kaplan–Meier curves in general and CKD populations, respectively. The statistical significance of differences among sub-groups was determined using the log-rank test. We employed the Cox proportional hazards regression model to assess the associations between Klotho and all-cause mortality in both general and CKD populations. The Schoenfeld residuals test was utilized to evaluate the validity of the proportional hazard assumption, and no significant violation was detected. A significance level of P < 0.05 (two-tailed) was considered statistically significant.

## Results

### *Klotho* gene is mainly expressed in tubulointerstitium and correlates well with renal function in CKD

**Fig. 1:**
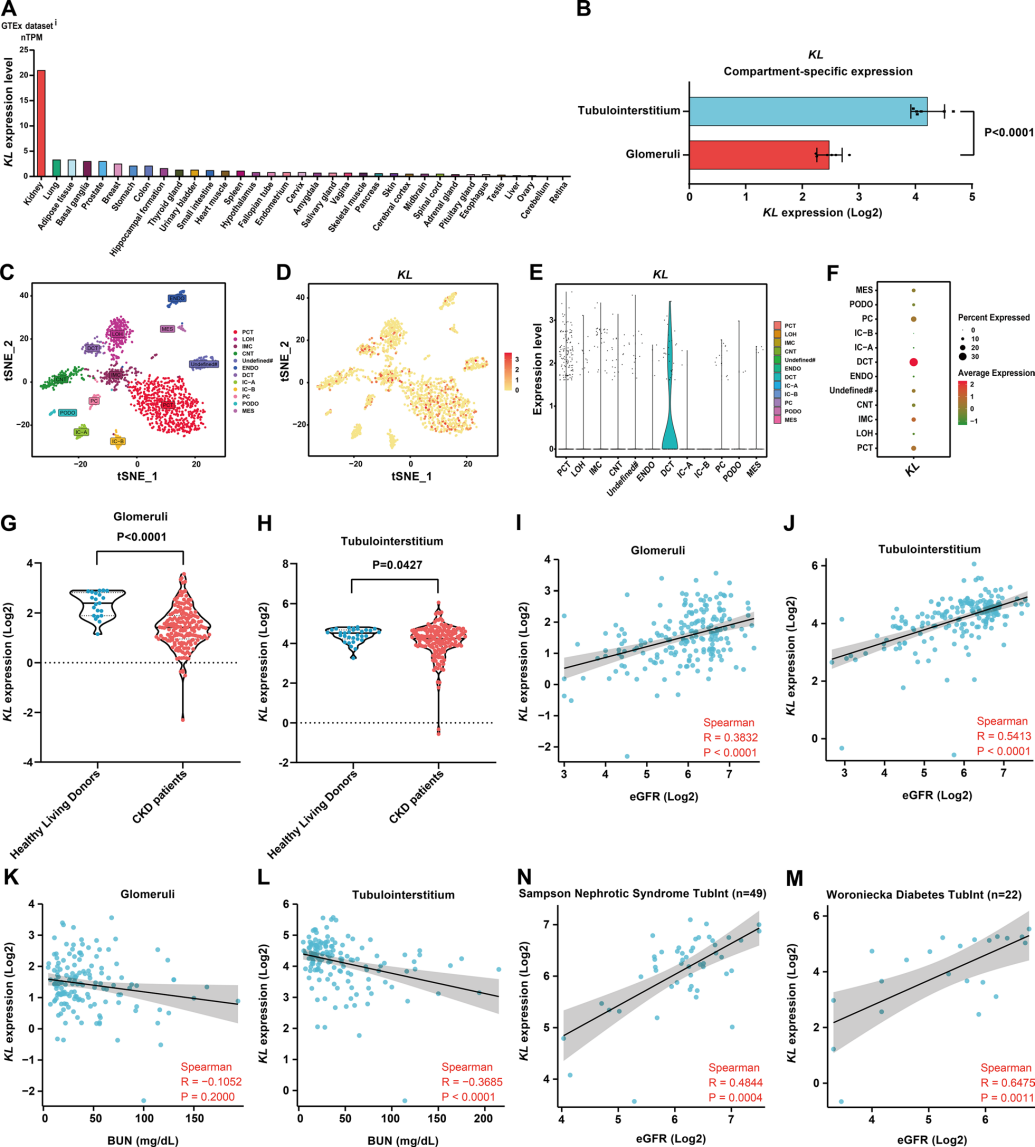
Analysis of *Klotho* gene expression in kidney and its relationship to renal function in CKD.** A** *KL* expression in major human organs/tissues displayed a highly kidney-specific expression pattern. **B** *KL* expression was prominently higher (fold change = 3.334, P < 0.0001) in tubulointerstitium (n = 6) compared to glomeruli (n = 6) based on t-test statistics. **C** The tSNE plot indicated that the clustering of snRNA-seq of adult human kidney identified 12 distinct cell types. That included proximal convoluted tubule (PCT), loop of Henle (LOH), distal convoluted tubule (DCT), connecting tubule (CNT), principal cell (PC), intercalated cell type A (IC-A), intercalated cell type B (IC-B), podocyte (PODO), endothelium (ENDO), mesangial cell (MES), Immune cell (IMC), and Undefined cell (Undefined#). **D**-**F** Expression of *KL* in 12 different cell types as demonstrated by tSNE plot, Violin plots and Dot plot. **G** *KL* expression of glomeruli in CKD patients (n = 172) and Healthy Living Donors (n = 21). **H** *KL* expression of tubulointerstitium in CKD patients (n = 170) and Healthy Living Donors (n = 31). **I** Correlation between *KL* expression of glomeruli and eGFR of CKD patients (n = 192). **J** Correlation between *KL* expression of tubulointerstitium and eGFR of CKD patients (n = 186). **K** Correlation between *KL* expression of glomeruli and BUN of CKD patients (n = 150). **L** Correlation between *KL* expression of tubulointerstitium and BUN of CKD patients (n = 142). **N**, **M** Correlation between *KL* expression of tubulointerstitium and eGFR of patients in an independent CKD cohort (Sampson Nephrotic Syndrome TubInt) and a DN cohort (Woroniecka Diabetes TubInt). *KL*, Klotho gene; eGFR, estimated glomerular filtration rate; BUN, blood urea nitrogen; CKD, chronic kidney disease; DN, diabetic nephropathy; Spearman, Spearman correlation analysis; R, correlation coefficient R; P, P-value

Given that the study reported that Klotho was mainly derived from the kidney [53], we analyzed the expression of *KL* in major organs/tissues throughout the body (data from the Human Protein Atlas database, Protein Atlas version 23.0) and found that the *Klotho* gene displayed a highly kidney-specific expression (Fig. 1A). We further analyzed *KL* expression in different compartments of normal kidney (data from the Nephroseq v5 database, raw data from GSE21785), and *KL* expression was significantly higher in the tubulointerstitium compared to the glomeruli (fold change = 3.334, P < 0.0001) (Fig. 1B). We also analyzed the expression of *KL* in normal adult kidney cells by snRNA-seq data (data from GSE118184) and found that *KL* was mainly expressed in distal convoluted tubule epithelial cells, followed by proximal convoluted tubule epithelial cells and also expressed in immune cells (Fig. 1C-F).

We also analyzed *KL* expression in the kidney of CKD and its relationship with renal function. Since the expression of *KL* in the glomeruli and tubulointerstitium differed significantly (Fig. 1B), we analyzed them separately (data from the Ju CKD Glom and Ju CKD TubInt datasets in the Nephroseq v5 database). We found that *KL* expression in both glomeruli and tubulointerstitium was significantly lower in CKD patients than in Healthy Living Donors (p < 0.0001 and P = 0.0427) (Fig. 1G, H). We further analyzed the relationship between glomerular and tubulointerstitial *KL* expression and eGFR or BUN in CKD by Spearman correlation analysis, respectively, and found that both glomerular and tubulointerstitial *KL* expressions in CKD were positively correlated with eGFR (R = 0.3832 and 0.5413, all P < 0.0001) (Fig. 1I, J). However, when analyzing the correlation between *KL* expression and BUN, only *KL* expression in the CKD tubulointerstitium was negatively correlated with BUN (R =−0.3685, P < 0.0001) (Fig. 1L), while *KL* expression in the CKD glomeruli was not correlated with BUN (R =−0.1052, P = 0.2000) (Fig. 1K). The results obtained indicate a stronger correlation between *KL* expression in the renal tubulointerstitium and renal function than in the glomeruli. We also analyzed two independent tubulointerstitial transcriptome datasets for CKD (data from the Nephroseq v5 database) and found that *KL* expression was positively correlated with eGFR in both the Sampson Nephrotic Syndrome TubInt and Woroniecka Diabetes TubInt datasets (R = 0.4844 and 0.6475, P = 0.0004 and 0.0011) (Fig. 1N, M), which further confirmed that *Klotho* gene expression in the tubulointerstitium was well correlated with renal function.

### *Klotho* gene expression is mainly negatively associated with the inflammatory response in CKD tubulointerstitium

**Fig. 2:**
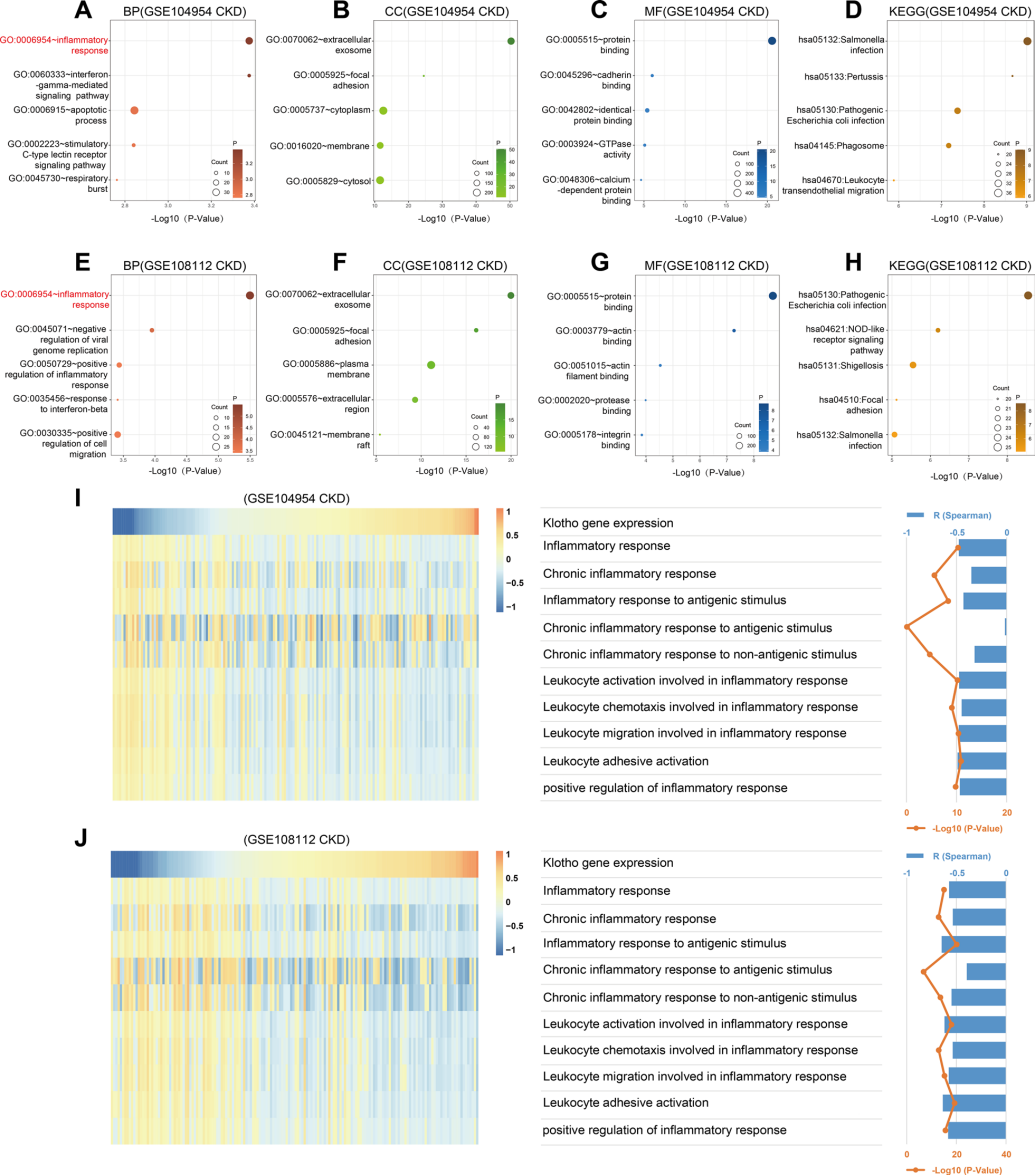
*Klotho* gene expression is predominantly negatively associated with the inflammatory response in CKD tubulointerstitium.** A**-**D** The top 5 biological processes (BP), cellular components (CC), molecular functions (MF), and Kyoto Encyclopedia of Genes and Genomes (KEGG) pathways were mostly related to the *Klotho* gene in the GSE104954 (CKD). **E**-**H** The top 5 biological processes (BP), cellular components (CC), molecular functions (MF), and Kyoto Encyclopedia of Genes and Genomes (KEGG) pathways were mostly related to the *Klotho* gene in the GSE108112 (CKD). **I**, **J** The heatmaps showed *Klotho* gene expression and enrichment scores of inflammatory response functions of each patient in the GSE104954 (CKD) and GSE108112 (CKD). The samples were arranged in ascending order of *Klotho* gene expression. The bar and point line plots on the right showed the correlation coefficient R and P-value of the Spearman correlation analysis

The *Klotho* gene is mainly expressed in the tubulointerstitium, and its expression correlates well with renal function in CKD. Therefore, we focused on its main regulatory pathways in the CKD tubulointerstitium. We first investigated its major negative regulatory pathway in CKD tubulointerstitium. We respectively used the GSE104954 (CKD) and GSE108112 (CKD) datasets to screen the genes that were significantly negatively associated with *KL* expression by Spearman correlation analysis (correlation coefficient R < -0.5, P < 0.05). Gene Ontology (GO) and Kyoto Encyclopedia of Genes and Genomes (KEGG) analyses were performed on the above screened genes in the DAVID database. In both the GSE104954 (CKD) and GSE108112 (CKD) datasets, the biological process most associated with *Klotho* gene is the inflammatory response (Fig. 2A, E), the cellular component most related to the *Klotho* gene is extracellular exosome (Fig. 2B, F), and the molecular function most associated with *Klotho* gene is protein binding (Fig. 2C, G). Both datasets in the KEGG analysis exhibited some pathways related to inflammation (Fig. 2D, H). The above results suggest that Klotho may negatively regulate the inflammatory response mainly through extracellular secretion and protein binding. We next used gene set variation analysis (GSVA) by GSE104954 (CKD) and GSE108112 (CKD) datasets to determine enrichment scores for inflammatory response processes. Correlation analysis between enrichment scores and *KL* expression showed that *Klotho* gene expression was negatively correlated with inflammatory response-related functions in both datasets (Fig. 2I, J), except in the GSE104954 (CKD) dataset, where there was no correlation with the chronic inflammatory response to antigenic stimulus (Fig. 2I). These results suggest that Klotho may mainly negatively regulate the inflammatory response in CKD tubulointerstitium.

### Klotho may affect immune cell infiltration in CKD tubulointerstitium

**Fig. 3:**
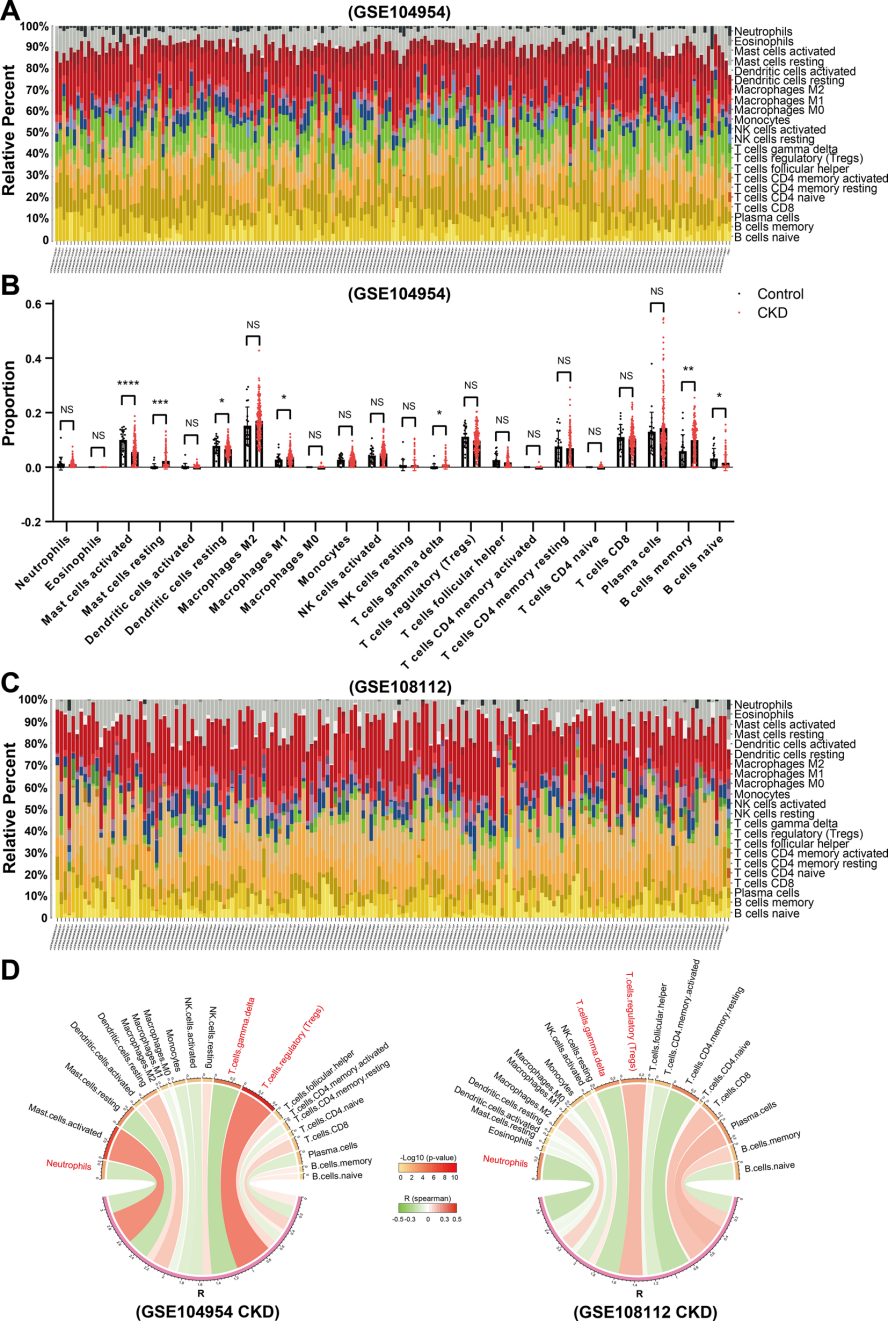
Immune cell infiltration analysis in CKD tubulointerstitium.** A**, **C** The percentage of 22 immune cells for each sample of GSE104954 and GSE108112. **B** Comparison of the proportion of each immune cell in the tubulointerstitium between Control and CKD groups. **D** Correlation between *Klotho* gene expression and the percentage of each immune cell of each sample in GSE104954 (CKD) and GSE108112 (CKD). The width of the band represented the correlation coefficient R in the chord diagram. The red band represented a positive correlation; the green band represented a negative correlation. The color of the upper outer band represented the P-value. The correlation was tested by Spearman correlation analysis. R represented correlation coefficient. *P < 0.05, **P < 0.01, ***P < 0.001, ****P < 0.0001

Given that the *Klotho* gene expression is mainly negatively associated with the inflammatory response in CKD tubulointerstitium, and soluble Klotho primarily originates from cleaved membrane-bound Klotho [54], exerting its influence on the physiological functions of neighboring cells through paracrine secretion, as well as the presence of immune cell infiltration in the CKD tubulointerstitium [55], we next investigated the relationship between Klotho and immune cell infiltration in CKD tubulointerstitium. We studied the percentage of 22 immune cells in the GSE104954 and GSE108112 datasets using the online tool CIBERSORTx (Fig. 3A, C) and found that in GSE104954, the percentage of Mast cells activated, Dendritic cells resting and B cells naive in CKD were significantly lower than those in normal controls (all P < 0.05), while the percentage of Mast cells resting, Macrophages M1, T cells gamma delta and B cells memory in CKD were significantly higher than those in normal controls (all P < 0.05) (Fig. 3B). These analyses were not performed in the GSE108112 dataset due to the small number of normal controls (5 cases). We next analyzed the correlation between the percentage of 21 immune cells and *Klotho* gene expression in GSE104954 (CKD) and GSE108112 (CKD) and found that the percentage of Neutrophils and T cells gamma delta was negatively correlated with *KL* expression in both GSE104954 (CKD) and GSE108112 (CKD) datasets, and T cells regulatory (Tregs) was positively correlated with *KL* expression in both datasets (all P < 0.05) (Fig. 3D). These findings suggest that Klotho may be involved in the regulation of immune cell infiltration in CKD tubulointerstitium.

To verify that Klotho may affect immune cell infiltration, we performed in vitro experiments. Considering that Klotho could modulate monocyte inflammatory factor release [56], and there was also a negative correlation between the percentage of monocytes in GSE104954 (CKD) and GSE108112 (CKD) and the expression of *KL* (with P < 0.05 in GSE108112) (Fig. 3D), we chose monocytes for the transwell invasion assay. Our results showed that Klotho could alleviate monocyte invasion under different induction factor conditions (Additional file 2: Fig. S1A, B), suggesting that Klotho may affect immune cell infiltration in CKD tubulointerstitium.

### *Klotho* gene expression is mainly positively associated with lipid metabolism in CKD tubulointerstitium

**Fig. 4:**
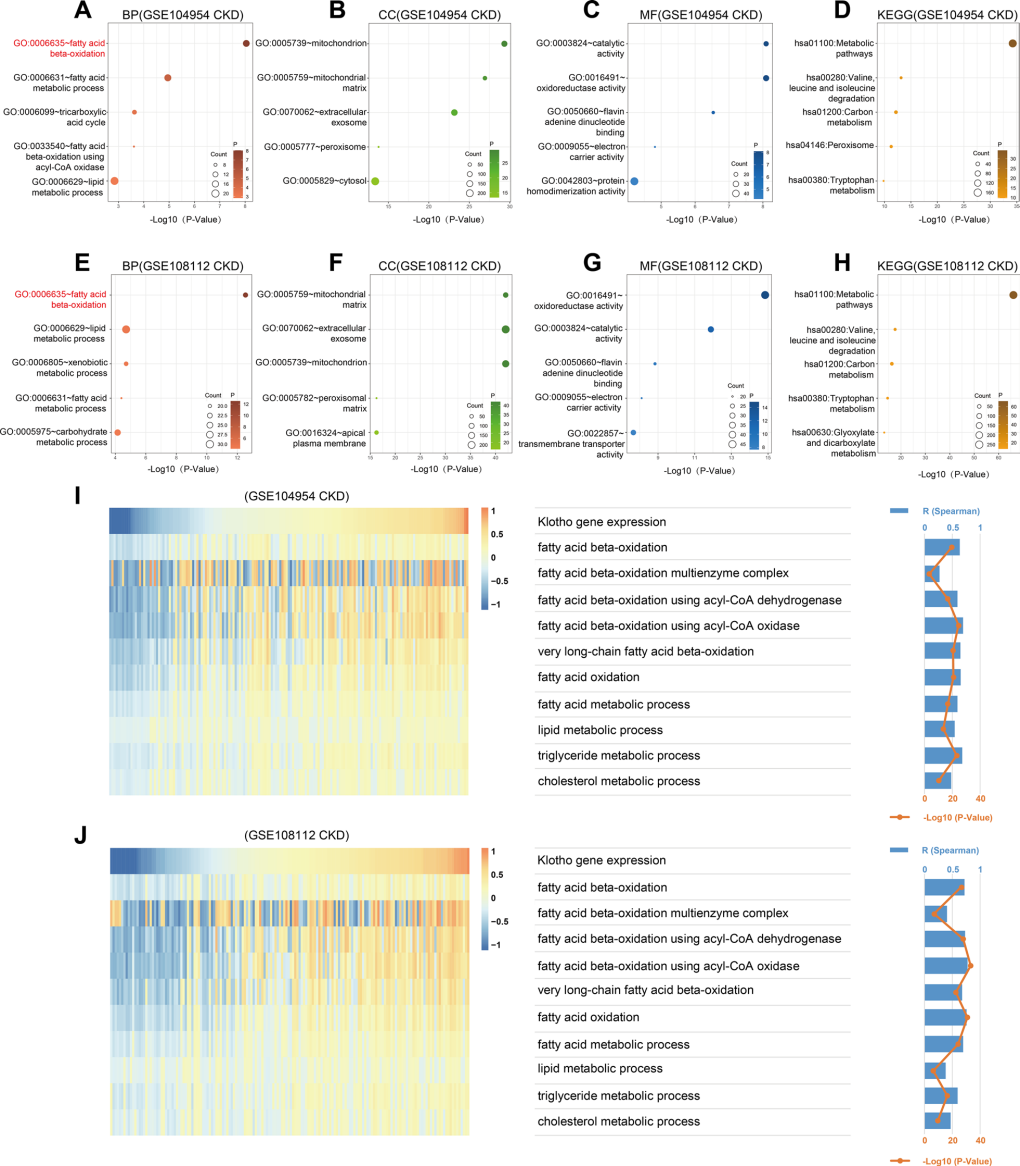
*Klotho* gene expression is predominantly positively associated with lipid metabolism in CKD tubulointerstitium.** A**-**D** The top 5 biological processes (BP), cellular components (CC), molecular functions (MF), and Kyoto Encyclopedia of Genes and Genomes (KEGG) pathways were mostly related to the *Klotho* gene in the GSE104954 (CKD). **E**-**H** The top 5 biological processes (BP), cellular components (CC), molecular functions (MF), and Kyoto Encyclopedia of Genes and Genomes (KEGG) pathways were mostly related to the *Klotho* gene in the GSE108112 (CKD). **I**, **J** The heatmaps showed *Klotho* gene expression and enrichment scores of lipid metabolism functions of each patient in the GSE104954 (CKD) and GSE108112 (CKD). The samples were arranged in ascending order of *Klotho* gene expression. The bar and point line plots on the right showed the correlation coefficient R and P-value of the Spearman correlation analysis

We have analyzed that the main negative regulatory pathway of Klotho in CKD tubulointerstitium may be the inflammatory response, but its main positive regulatory pathway is still unclear. Therefore, we also respectively used the GSE104954 (CKD) and GSE108112 (CKD) datasets to screen the genes that were significantly positively associated with *KL* expression by Spearman correlation analysis (correlation coefficient R > 0.5, P < 0.05). GO and KEGG analyses were performed on the above screened genes in the DAVID database. In the GSE104954 (CKD) and GSE108112 (CKD) datasets, the most relevant biological process with *Klotho* gene is fatty acid β-oxidation, followed by fatty acid metabolic process and lipid metabolic process (Fig. 4A, E), and the cellular components related to the *Klotho* gene are mitochondrion, mitochondrial matrix and extracellular exosome (Fig. 4B, F), and the molecular functions associated with *Klotho* gene are catalytic activity, oxidoreductase activity, flavin adenine dinucleotide binding, and electron carrier activity (Fig. 4C, G). Both datasets in the KEGG analysis show mainly metabolic pathways (Fig. 4D, H). The above results suggest that Klotho may positively regulate lipid metabolism mainly through the involvement of cellular mitochondrion in fatty acid β-oxidation. We next used gene set variation analysis (GSVA) in the GSE104954 (CKD) and GSE108112 (CKD) datasets to determine enrichment scores for lipid metabolism. Correlation analysis between enrichment scores and *KL* expression showed a positive correlation between *Klotho* gene expression and lipid metabolism-related functions in both datasets (Fig. 4I, J). These results suggest that Klotho may mainly positively regulate lipid metabolism in CKD tubulointerstitium.

### Klotho may improve fatty acid oxidation by regulating *PPARA* and *PPARGC1A* expression in CKD tubulointerstitium

**Fig. 5:**
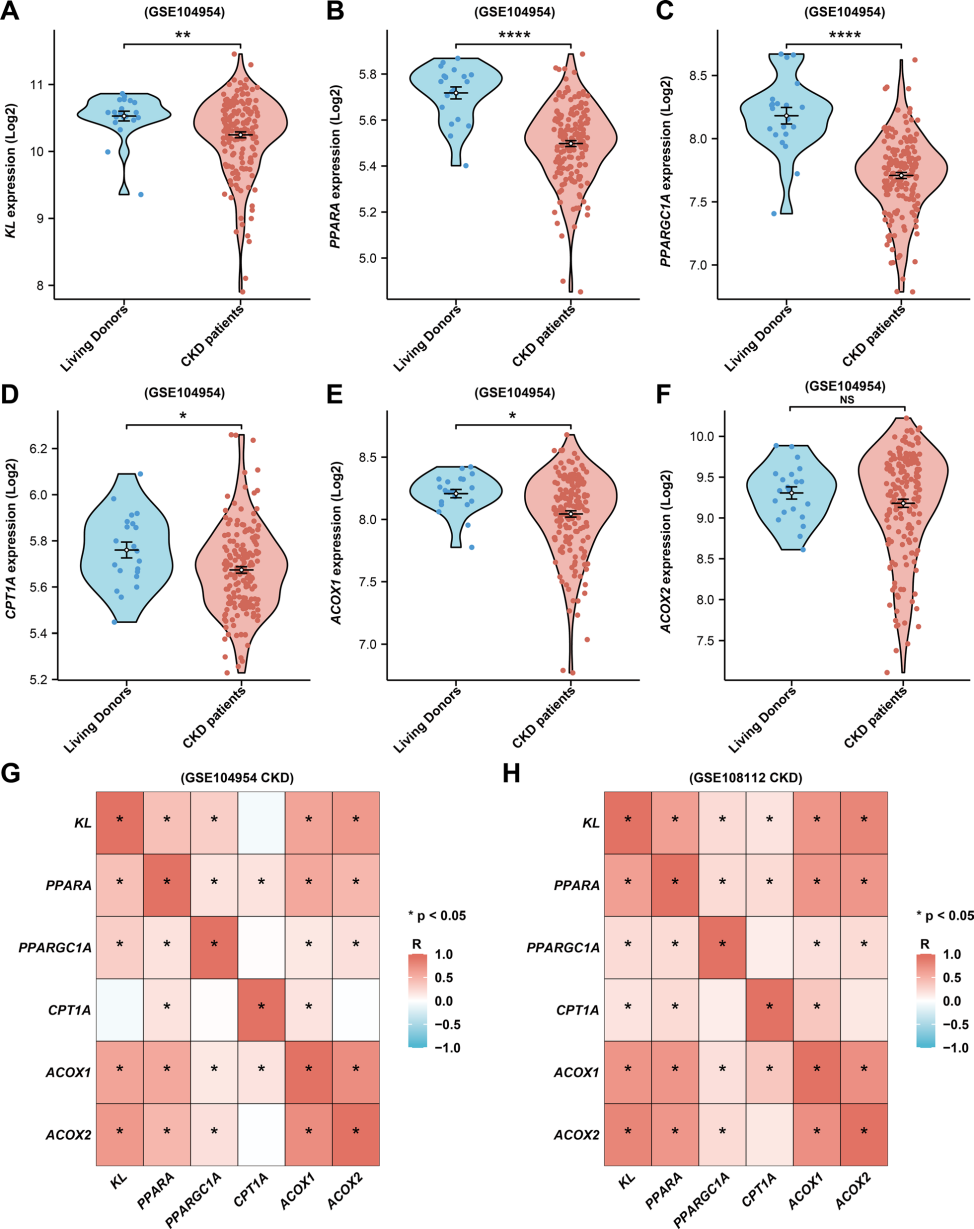
Relationship analysis of *Klotho* gene expression with fatty acid oxidation-related genes expression in CKD tubulointerstitium.** A**-**F** Comparative analysis of the expression of *KL*, *PPARA*, *PPARGC1A*, *CPT1A*, *ACOX1* and *ACOX2* in tubulointerstitium of Living Donors (n = 21) and CKD patients (n = 169) based on the Mann-Whitney test in GSE104954. **G**, **H** The heatmaps showed the correlation between each of genes (*KL*, *PPARA*, *PPARGC1A*, *CPT1A*, *ACOX1* and *ACOX2*) in GSE104954 (CKD) and GSE108112 (CKD). The correlation was tested using Spearman correlation analysis. R represented correlation coefficient. *P < 0.05, **P < 0.01, ****P < 0.0001

**Fig. 6:**
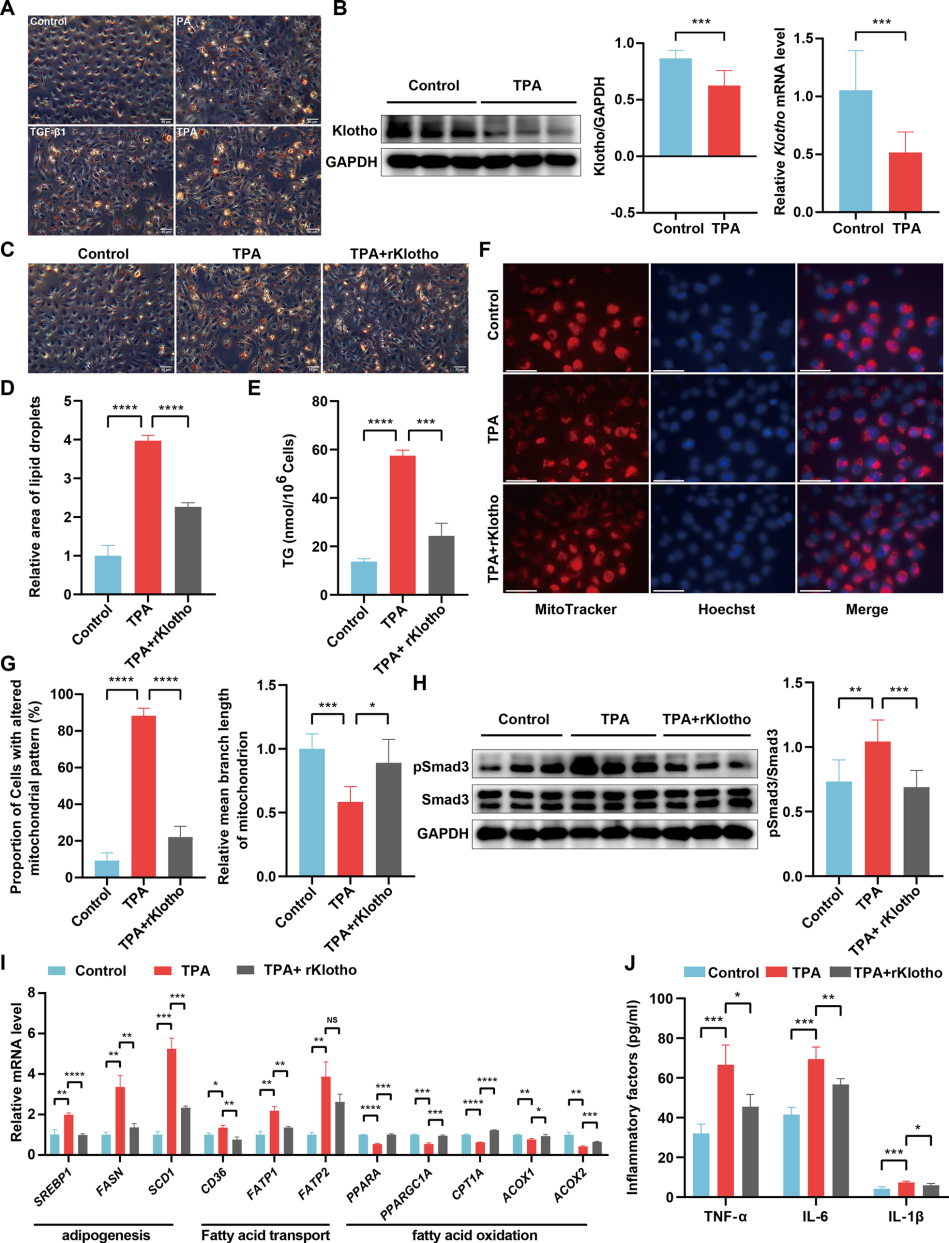
Klotho reduced inflammatory response and improved lipid metabolism in HK-2 treated with TPA. **A** Representative photomicrographs of Oil Red O staining of HK-2 treated with PA, TGF-β1, and TPA. Scale bars = 50 μm. **B** Western blot and real-time PCR analysis of Klotho expression in HK-2 treated with TPA. The molecular weight of Klotho was about 62 kDa, representing soluble Klotho in the Western blot bands. The molecular weight of GAPDH was 36 kDa. **C** Representative photomicrographs of Oil Red O staining of HK-2 treated with TPA and TPA + rKlotho. Scale bars = 50 μm. **D** Statistical analysis of lipid droplets in each group in **C**. **E** Detection of TG concentration in HK-2 treated with TPA and TPA + rKlotho. **F** Representative photomicrographs of mitochondria labeled with MitoTracker in HK-2 treated with TPA and TPA + rKlotho. Nuclei was stained with Hoechst (blue). Scale bars = 60 μm. **G** Statistical analysis of percentage of cells with altered mitochondrial pattern and mean branch length of mitochondrion in each group in **F**. **H** Western blot analysis of pSmad3 and Smad3 expression in HK-2 treated with TPA and TPA + rKlotho. The molecular weight of pSmad3 and Smad3 was 55 kDa. **I** Real-time PCR analysis of *SREBP1*, *FASN*, *SCD1*, *CD36*, *FATP1*, *FATP2*, *PPARA*, *PPARGC1A*, *CPT1A*, *ACOX1* and *ACOX2* expression in HK-2 treated with TPA and TPA + rKlotho. **J** The concentrations of TNF-α, IL-6, and IL-1β in the culture supernatants of HK-2 treated with TPA and TPA + rKlotho. PA, palmitic acid; TPA, TGF-β1 and palmitic acid; rKlotho, recombinant human Klotho; TG, triglyceride. The results were mean ± SEM of three independent experiments. *P < 0.05, ** P < 0.01, ***P < 0.001, ****P < 0.0001

Peroxisome proliferator-activated receptor α (*PPARA*) and PPAR-γ coactivator-1α (*PPARGC1A*) are the key transcription factors that regulate the expression of fatty acid oxidation-related enzymes [57], so we analyzed the expression of *PPARA*, *PPARGC1A*, fatty acid oxidation-related enzyme genes and *KL* in GSE104954. We found that *KL* expression was significantly decreased in CKD patients compared to Living Donors (P < 0.01) (Fig. 5A), which is consistent with the former result (Fig. 1H). It was also found that the expression of *PPARA*, *PPARGC1A*, *CPT1A* and *ACOX1* was also significantly decreased in CKD patients compared to Living Donors (all P < 0.05) (Fig. 5B-E). These results suggest that fatty acid oxidation may be hindered in CKD tubulointerstitium. We further analyzed the correlation between *KL* expression and the expression of the above fatty acid oxidation-related genes. We found that except for *CPT1A* expression, which showed no significant correlation with *KL* expression in GSE104954 (CKD), the remaining genes showed a clear positive correlation with *KL* expression in both GSE104954 (CKD) and GSE108112 (CKD) (Fig. 5G, H). Moreover, our in vitro experiments also confirmed that Klotho could positively regulate the expression of *PPARA*, *PPARGC1A*, *CPT1A*, *ACOX1* and *ACOX2* in HK-2 treated with TPA (Fig. 6I). In particular, we also demonstrated at the protein level that Klotho could positively regulate the expression of PPARα and PGC1α (Additional file 2: Fig. S2A-C). Given that Klotho ameliorated abnormal lipid metabolism in the liver by upregulating *PPARA* expression in type 2 diabetic mice [14], our results suggest that Klotho may improve fatty acid oxidation by regulating *PPARA* and *PPARGC1A* expression.

### Klotho regulated inflammatory response and lipid metabolism in HK-2 exposed to a CKD-like environment

We have found that Klotho may mainly negatively regulate inflammatory response and positively regulate lipid metabolism in CKD tubulointerstitium by bioinformatics analysis. Next, we sought to confirm our findings by establishing an in vitro model similar to CKD. We found that HK-2 showed intracellular lipid droplets accumulation after 48 h of treatment with either palmitic acid or TGF-β1, especially after HK-2 was treated with both palmitic acid and TGF-β1 (Fig. 6A). Given that lipids and TGF-β1 are elevated in CKD [58, 59], we constructed an in vitro model similar to CKD by simultaneously stimulating HK-2 with TGF-β1 and palmitic acid (TPA). We found that TPA significantly decreased the expression of Klotho and increased the accumulation of lipid droplets and TG content compared to controls in HK-2 (Fig. 6B-E). However, the addition of rKlotho treatment markedly reduced the accumulation of lipid droplets and TG content compared to TPA groups in HK-2 (Fig. 6C-E). We found that the involvement of Klotho in fatty acid β-oxidation might be associated with mitochondrion (Fig. 4B, F), so we observed the morphological changes of mitochondria with MitoTracker in HK-2 treated with TPA. We found that TPA significantly altered mitochondrial morphology and shortened the average branch length of each mitochondrion, but the addition of rklotho reversed the above process (Fig. 6F, G).

Given that TGF-β1 depends on Smad3 to repress fatty acid oxidation [57], and the activation of Smad3 also mediates inflammation [60], we examined the expression of pSmad3 and Smad3 in HK-2 treated with TPA. We observed that TPA significantly activated the phosphorylation of Smad3 (pSmad3), but this was obviously inhibited by the addition of rKlotho (Fig. 6H). We next examined the transcript levels of genes related to fatty acid oxidation and found that TPA significantly inhibited the expression of *PPARA*, *PPARGC1A*, *CPT1A*, *ACOX1* and *ACOX2*, but was significantly improved by the addition of rKlotho (Fig. 6I). Moreover, rKlotho also markedly downregulated the abnormal elevation of the adipogenic genes (*SREBP1*, *FASN* and *SCD1*) and fatty acid transporter genes (*CD36* and *FATP1*) in HK-2 treated with TPA (Fig. 6I). Finally, we found that TPA induced the HK-2 inflammatory response to release the inflammatory factors TNF-α, IL-6 and IL-1β, but was inhibited by rKlotho (Fig. 6J). Together, these results confirmed that Klotho could reduce the inflammatory response and improve lipid metabolism in HK-2 exposed to a CKD-like environment.

**Fig. 7:**
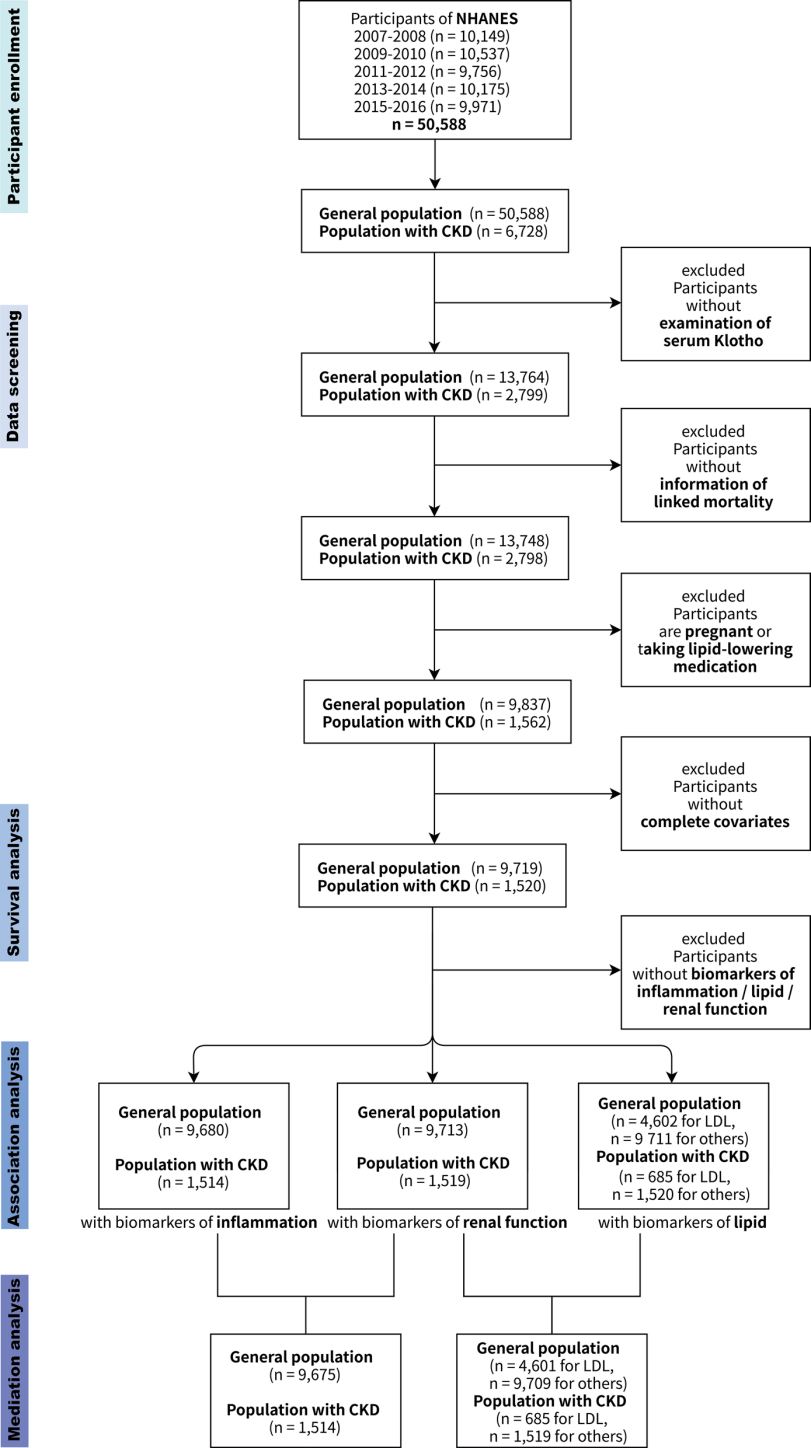
Flowchart of studying the NHANES 2007–2016 Klotho-related clinical data. The others include high density lipoprotein (HDL), total cholesterol (TC) and triglyceride (TG). CKD, chronic kidney disease; LDL, low density lipoprotein

Based on all the results above, we have demonstrated a good correlation between *Klotho* and renal function at the gene level through bioinformatics analysis. In particular, we have found that inflammatory response and lipid metabolism are the top pathways that Klotho may regulate in CKD tubulointerstitium. Moreover, our in vitro experiments also confirmed that Klotho could negatively regulate inflammatory response and positively regulate lipid metabolism. Given that a landmark study shows that inflammation and metabolism are the primary dysregulated pathways in CKD tubulointerstitium [57], in which metabolic abnormalities mainly include abnormal lipid metabolism, coupled with a parallel relationship between soluble Klotho levels in peripheral blood and renal Klotho expression levels [2], as well as dyslipidemia and elevated inflammatory markers in CKD [61, 62], we speculate that there is a close relationship between Klotho and blood lipids, inflammatory markers and kidney function in the CKD population. Therefore, we next used the clinical data from NHANES 2007–2016 containing the Klotho indicator to confirm our speculation. See Fig. 7 for the research flowchart.

### Characteristics of NHANES population

Among the 9719 adults (aged 40–79 years) from five NHANES cycles, 1520 participants were diagnosed with CKD, and the remainder were participants without CKD. Lipid biomarkers, inflammatory biomarkers and renal function biomarkers were described based on the actual number of participants with these biomarkers in the non-CKD and CKD populations. The baseline characteristics of study participants with and without CKD were summarized in Table 1. Overall, there were statistically significant differences between non-CKD and CKD participants in terms of sex, age, race, educational attainment, BMI, smoking status, CVD, DM, hypertension, Klotho, TC, TG, WBC, Neu, Mono, SII, NLR, MLR, SIRI, PIV, eGFR, serum creatinine, uric acid and serum urea nitrogen (all P < 0.05). However, no significant differences were observed between the two groups for other variables, including HDL, LDL, Lym, PLR and UACR (all P > 0.05).

**Table 1:** Descriptive characteristics of NHANES participants

Characteristics	General population	Population without CKD	Population with CKD	P-value
Sex				**0.003**
Male	4543 (46.74)	3870 (46.35)	673 (39.91)	
Female	5176 (53.26)	4329 (53.65)	847 (60.09)	
Age in years	53.96 (0.16)	53.11 (0.17)	59.81 (0.33)	** < 0.001**
Age				** < 0.001**
40–50	3445 (35.45)	3139 (41.58)	306 (22.25)	
50–60	2799 (28.8)	2453 (33.80)	346 (25.63)	
60–70	2341 (24.09)	1871 (18.02)	470 (29.14)	
> = 70	1134 (11.67)	736 (6.60)	398 (22.97)	
Race				** < 0.001**
Mexican American	1643 (16.91)	1380 (7.29)	263 (8.09)	
Non-Hispanic White	4002 (41.18)	3400 (71.88)	602 (67.58)	
Non-Hispanic Black	1925 (19.81)	1559 (9.01)	366 (12.62)	
Other Races	2149 (22.11)	1860 (11.83)	289 (11.71)	
Educational attainment				** < 0.001**
< 11th grade	2683 (27.61)	2177 (15.44)	506 (21.18)	
High school graduate	2135 (21.97)	1785 (21.71)	350 (23.65)	
Some college or AA degree	2653 (27.3)	2253 (30.48)	400 (30.05)	
College graduate or above	2248 (23.13)	1984 (32.37)	264 (25.13)	
BMI				** < 0.001**
Normal	2595 (26.7)	2258 (28.33)	337 (22.65)	
Overweight	3382 (34.8)	2913 (35.92)	469 (31.21)	
Obese	3742 (38.5)	3028 (35.75)	714 (46.13)	
Smoking status				**0.001**
Never	5182 (53.32)	4436 (54.41)	746 (48.26)	
Former	2485 (25.57)	2059 (26.21)	426 (31.13)	
Current	2052 (21.11)	1704 (19.38)	348 (20.60)	
CVD				** < 0.001**
Yes	721 (7.42)	472 (4.44)	249 (12.87)	
No	8998 (92.58)	7727 (95.56)	1271 (87.13)	
DM				** < 0.001**
Yes	1670 (17.18)	1131 (9.87)	539 (28.48)	
No	8049 (82.82)	7068 (90.13)	981 (71.52)	
Hypertension				** < 0.001**
Yes	4386 (45.13)	3343 (36.94)	1043 (63.34)	
No	5333 (54.87)	4856 (63.06)	477 (36.66)	
Klotho (pg/ml)	859.53 (5.97)	863.18 (6.36)	834.54 (9.88)	**0.008**
Biomarkers of lipid				
HDL (mg/dl)	55.33 (0.35)	55.42 (0.34)	54.70 (1.01)	0.464
LDL (mg/dl)	126.38 (0.74)	126.77 (0.75)	123.52 (1.92)	0.094
TC (mg/dl)	209.94 (0.75)	209.55 (0.75)	212.68 (1.63)	**0.047**
TG (mg/dl)	162.13 (2.16)	159.07 (2.34)	183.05 (4.67)	** < 0.001**
Biomarkers of inflammation				
WBC (1000 cells/ul)	6.57 (0.06)	6.51 (0.06)	7.01 (0.11)	** < 0.001**
Neu (1000 cell/ul)	3.87 (0.04)	3.82 (0.04)	4.27 (0.09)	** < 0.001**
Lym (1000 cell/ul)	1.92 (0.02)	1.92 (0.02)	1.92 (0.05)	0.966
Mono (1000 cell/ul)	0.53 (0.01)	0.52 (0.01)	0.56 (0.01)	**0.004**
SII	527.75 (8.13)	517.70 (8.20)	601.61 (19.63)	** < 0.001**
NLR	2.17 (0.03)	2.12 (0.03)	2.49 (0.07)	** < 0.001**
MLR	0.29 (0.00)	0.29 (0.00)	0.32 (0.01)	** < 0.001**
PLR	136.50 (1.39)	136.20 (1.45)	138.76 (2.28)	0.269
SIRI	1.17 (0.02)	1.14 (0.02)	1.43 (0.05)	** < 0.001**
PIV	288.06 (5.47)	279.62 (5.64)	350.07 (15.86)	** < 0.001**
Biomarkers of renal function				
eGFR (mL/min/1.73m^2^)	89.44 (0.35)	91.59 (0.31)	74.72 (0.90)	** < 0.001**
Serum creatinine (mg/dl)	0.87 (0.00)	0.84 (0.00)	1.08 (0.02)	** < 0.001**
UACR (mg/g)	16.09 (0.14)	16.09 (0.14)	16.10 (0.22)	0.937
Uric acid (mg/dl)	5.40 (0.03)	5.33 (0.03)	5.94 (0.06)	** < 0.001**
Serum urea nitrogen (mg/dl)	13.58 (0.10)	13.19 (0.09)	16.25 (0.25)	** < 0.001**

*CKD,* chronic kidney disease; *BMI*, body mass index; *CVD,* cardiovascular disease; *DM,* diabetes mellitus; *HDL,* high density lipoprotein; *LDL,* low density lipoprotein; *TC,* total cholesterol; *TG,* triglyceride; *WBC*, white blood cell; *Neu,* neutrophil; *Lym,* lymphocyte; *Mono,* monocyte; *SII,* systemic immune-inflammation index; *NLR,* neutrophil to lymphocyte ratio; *MLR,* monocyte to lymphocyte ratio; *PLR,* platelet to lymphocyte ratio; *SIRI*, systemic inflammatory response index; *PIV,* pan-immune-inflammation value; *eGFR,* estimated glomerular filtration rate; *UACR,* Urine Albumin-to-Creatinine Ratio. Continuous variables were presented as Mean ± SD, and categorical variables were presented as N (%)

### Klotho was negatively associated with inflammatory response and positively associated with lipid metabolism and renal function in general population

In the general population, the associations of Klotho with inflammatory biomarkers, lipid biomarkers, and renal function in both categorical and continuous analyses in generalized linear model (GLM) were presented in Additional file 1: Table S2. Using the lowest tertile of Klotho level (T1) as the reference, the β and 95% CI for TC, eGFR, serum urea nitrogen, serum creatinine, and uric acid in the moderate tertile of Klotho level (T2) were β − 3.227 (95% CI − 6.288, − 0.167), β 1.689 (95% CI 0.596, 2.782), β − 0.409 (95% CI − 0.668, − 0.150), β − 0.033 (95% CI − 0.050, − 0.016) and β − 0.172 (95% CI − 0.255, − 0.088) in the adjusted model (all P < 0.05). Furthermore, using the T1 as the reference, the β and 95% CI for WBC, Neu, PLR, PIV, SII, TC, TG, eGFR, serum urea nitrogen, serum creatinine and uric acid in the highest tertile of Klotho level (T3) were β − 0.174 (95% CI − 0.319, − 0.028), β − 0.160 (95% CI − 0.288, − 0.032), β − 5.865 (95% CI − 9.028, − 2.702), β − 27.806 (95% CI − 43.335, − 12.277), β −43.053 ( 95% CI − 65.437, − 20.669), β − 4.335 (95% CI − 7.629, − 1.041), β − 12.756 (95% CI − 23.960, − 1.552), β 2.799 (95% CI 1.792, 3.806), β − 0.589 (95% CI − 0.892, − 0.285), β − 0.050 (95% CI − 0.068, − 0.032) and β − 0.351 (95% CI − 0.434, − 0.269) in the adjusted model (all P < 0.05). The continuous analysis showed that Klotho had a significant negative association with WBC, Neu, NLR, PLR, PIV, SIRI, SII, TC, TG, serum urea nitrogen, serum creatinine and uric acid, and a positive association with eGFR (all P < 0.05, adjusted model).

### Klotho was negatively associated with inflammatory response and positively associated with lipid metabolism and renal function in CKD population

In the CKD population, the associations of Klotho with inflammatory biomarkers, lipid biomarkers, and renal function in both categorical and continuous analyses in GLM were presented in Additional file 1: Table S3. Using the T1 as the reference, the β and 95% CI for WBC, Neu, Mono, SIRI, TG, eGFR, serum urea nitrogen and serum creatinine in the T2 were β − 0.365 (95% CI − 0.665, − 0.065), β − 0.293 (95% CI − 0.548, − 0.038), β − 0.033 (95% CI − 0.064, − 0.002), β − 0.178 (95% CI − 0.346, − 0.009), β − 22.273 (95% CI − 43.217, − 1.329), β 5.917 (95% CI 1.772, 10.062), β − 1.586 (95% CI − 2.562, − 0.611) and β − 0.167 (95% CI − 0.270, − 0.065) in the adjusted model (all P < 0.05). Furthermore, using the T1 as the reference, the β and 95% CI for WBC, Neu, NLR, MLR, PLR, PIV, SIRI, SII, TG, eGFR, serum urea nitrogen, serum creatinine and uric acid in the T3 were β − 0.414 (95% CI − 0.685, − 0.143), β − 0.399 (95% CI − 0.668, − 0.129), β − 0.275 (95% CI − 0.473, − 0.077), β − 0.026 (95% CI − 0.047, − 0.006), β − 7.627 (95% CI − 15.041, − 0.212), β − 74.675 (95% CI − 123.447, − 25.904), β − 0.237 (95% CI − 0.415, − 0.060), β − 97.05 (95% CI − 149.552, − 44.548), β − 21.19 (95% CI − 41.948, − 0.432), β 8.476 (95% CI 5.046, 11.906), β − 2.499 (95% CI − 3.451, − 1.548), β − 0.231 (95% CI − 0.343, − 0.119) and β − 0.581 (95% CI − 0.829, − 0.332) in the adjusted model (all P < 0.05). The continuous analysis showed that Klotho had a significant negative association with WBC, Neu, NLR, MLR, PLR, PIV, SIRI, SII, serum urea nitrogen, serum creatinine and uric acid, and a positive association with eGFR (all P < 0.05, adjusted model).

### Klotho has an optimal concentration range for exerting its biological function

**Fig. 8:**
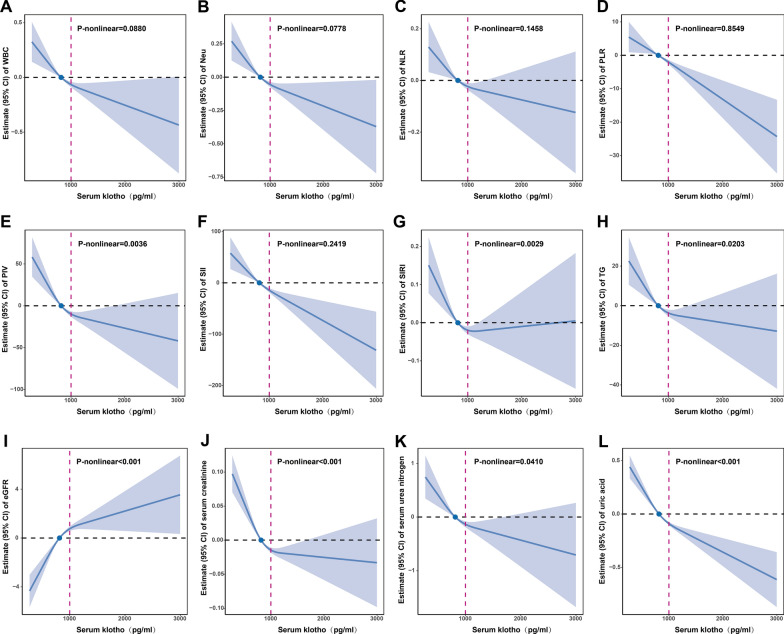
RCS estimated the dose–response relationships between Klotho and 95% CIs of biomarkers in general population. The model was fully adjusted for sex, age, race, educational attainment, BMI, smoking status, CVD, DM and hypertension. RCS, restricted cubic spline; 95% CI, 95% confidence interval; Blue solid line, beta value; Blue dot, the median level of Klotho; Black dashed line, reference level; Red dashed line, Klotho 1000 pg/ml level; Blue shade, 95% CI

**Fig. 9:**
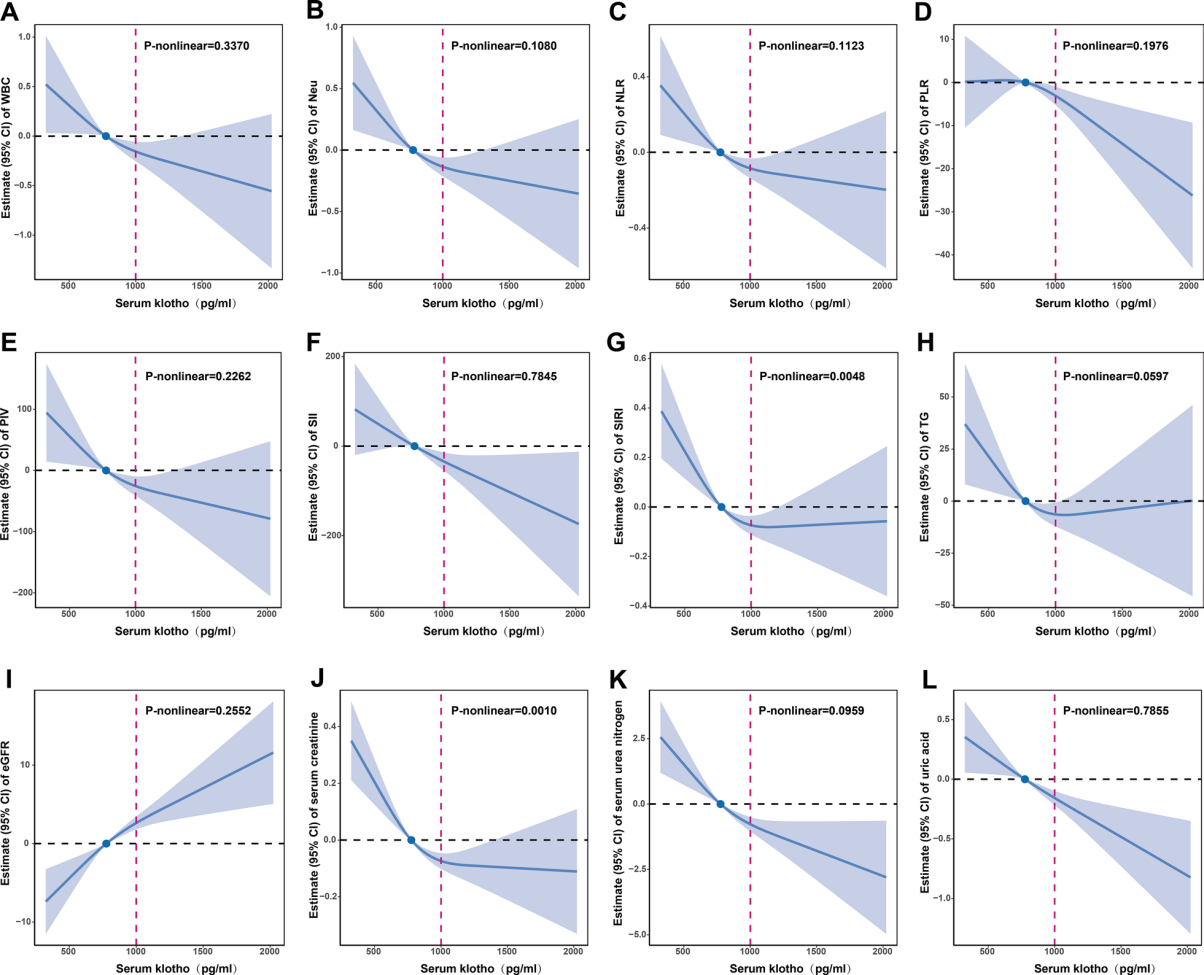
RCS estimated the dose–response relationships between Klotho and 95% CIs of biomarkers in CKD population. The model was fully adjusted for sex, age, race, educational attainment, BMI, smoking status, CVD, DM and hypertension. RCS, restricted cubic spline; 95% CI, 95% confidence interval; Blue solid line, beta value; Blue dot, the median level of Klotho; Black dashed line, reference level; Red dashed line, Klotho 1000 pg/ml level; Blue shade, 95% CI

Based on the fact that Klotho was negatively associated with WBC, Neu, NLR, PLR, PIV, SII, SIRI, TG, serum creatinine, serum urea nitrogen and uric acid, and positively associated with eGFR in the general and CKD populations (Additional file 1: Tables S2, S3), RCS was applied to examine the nonlinear relationship and threshold effect of Klotho with them. We found Klotho was linearly related to WBC, Neu, NLR, PLR and SII (all P-nonlinear > 0.05) and non-linearly related to PIV, SIRI, TG, eGFR, serum creatinine, serum urea nitrogen and uric acid (all P-nonlinear < 0.05) in the general population (Fig. 8). In the CKD population (Fig. 9), Klotho was linearly related to WBC, Neu, NLR, PLR, PIV, SII, TG, eGFR, serum urea nitrogen and uric acid (all P-nonlinear > 0.05) and non-linearly related to SIRI and serum creatinine (all P-nonlinear < 0.05). Except for indicators with P-nonlinear > 0.7, we found that the threshold effects of Klotho on the above 12 biological indicators were all close to 1000 pg/ml. The functional effects of Klotho were significantly altered in this threshold range (Figs. 8, 9). These results suggest that the optimal concentration range for Klotho to exert its biological function is around 1000 pg/ml.

### Biomarkers of inflammation and lipid mediated the association between Klotho and renal function in general population

To assess whether the association between Klotho and renal function may be mediated by biomarkers of inflammation and lipid in the general population, we performed a mediation analysis. In the general population (Additional file 1: Table S4), the results showed that inflammatory biomarkers (WBC, Neu, NLR, SIRI and SII) mediated the association between Klotho and eGFR, and the proportions were 3.749%, 3.747%, 2.073%, 1.448% and 2.375%, respectively (all p < 0.05). No significant mediation effects of lipid biomarkers (TC, TG, HDL and LDL) were observed in the association of Klotho with eGFR (all p > 0.05). Inflammatory biomarkers (WBC and Neu) mediated the association between Klotho and serum urea nitrogen, and the proportions were 3.594% and 2.468% (all p < 0.05). TG mediated the association between Klotho and serum urea nitrogen, and the proportion was 3.382% (p = 0.016). Inflammatory biomarkers (WBC, Neu, NLR, PIV, SIRI and SII) mediated the association between Klotho and serum creatinine, and the proportions were 2.482%, 3.158%, 2.808%, 1.913%, 1.801% and 3.113%, respectively (all p < 0.05). No significant mediation effects of lipid biomarkers (TC, TG, HDL and LDL) were observed in the association of Klotho with serum creatinine (all p > 0.05). Inflammatory biomarkers (WBC, Neu and SIRI) mediated the association between Klotho and uric acid, and the proportions were 2.134%, 1.749% and 0.688%, respectively (all p < 0.05). TC and TG mediated the association between Klotho and uric acid, and the proportions were 4.349% and 2.511% (all p < 0.05). No significant mediation effects of inflammatory biomarkers and lipid biomarkers were observed in the association of Klotho with UACR (all p > 0.05). These above results indicate that Klotho may improve renal function in the general population by regulating the inflammatory response and lipid metabolism.

### Inflammatory biomarkers mediated the association between Klotho and renal function in CKD population

In the CKD population (Additional file 1: Table S5), our results showed that inflammatory biomarkers (WBC and Neu) mediated the association between Klotho and eGFR, and the proportions were 3.478% and 3.449% (all p < 0.05). No significant mediation effects of lipid biomarkers (TC, TG, HDL and LDL) were observed in the association of Klotho with eGFR (all p > 0.05). WBC mediated the association between Klotho and serum urea nitrogen, and the proportion was 2.858% (p = 0.048). No significant mediation effects of lipid biomarkers (TC, TG, HDL and LDL) were observed in the association of Klotho with serum urea nitrogen (all p > 0.05). Inflammatory biomarkers (MLR and SIRI) mediated the association between Klotho and serum creatinine, and the proportions were 2.425% and 2.447% (all p < 0.05). No significant mediation effects of lipid biomarkers (TC, TG, HDL and LDL) were observed in the association of Klotho with serum creatinine (all p > 0.05). Inflammatory biomarkers (MLR, PIV and SII) mediated the association between Klotho and uric acid, and the proportions were 3.181%, 3.396% and 3.412%, respectively (all p < 0.05). No significant mediation effects of lipid biomarkers (TC, TG, HDL and LDL) were observed in the association of Klotho with uric acid (all p > 0.05). No significant mediation effects of inflammatory biomarkers and lipid biomarkers were observed in the association of Klotho with UACR (all p > 0.05). These above results suggest that Klotho may improve renal function in the CKD population by mainly regulating the inflammatory response.

### Klotho could reduce the risk of all-cause mortality in general and CKD populations

**Fig. 10:**
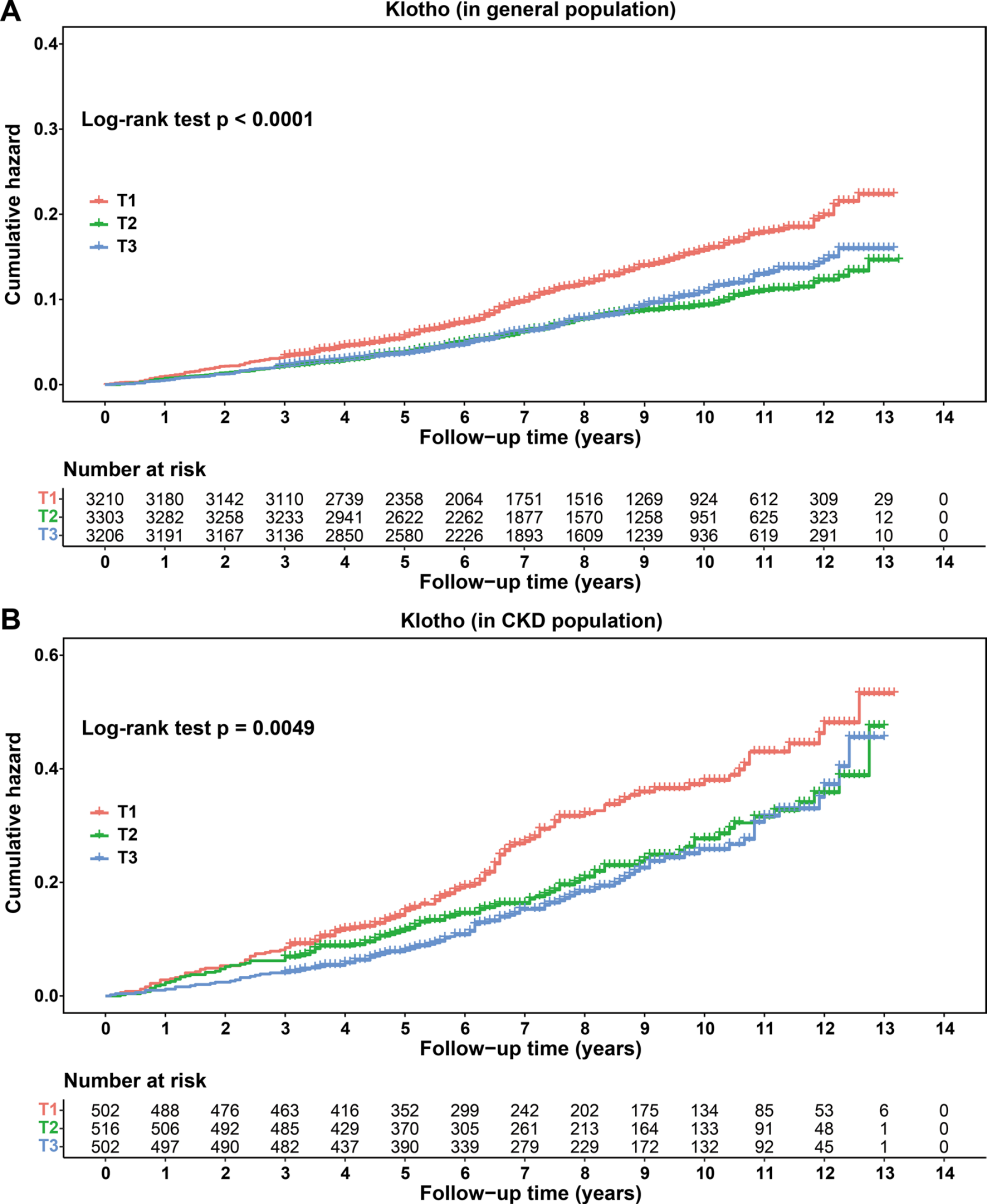
Kaplan–Meier curves of cumulative hazards. The general population **A** and CKD population **B** for risk of all-cause mortality by serum Klotho tertile level. T1, the lowest tertile of Klotho level; T2, the moderate tertile of Klotho level; T3, the highest tertile of Klotho level; P, P-value

Kaplan–Meier curves demonstrated the higher cumulative hazard of all-cause mortality in participants with the lowest tertile of Klotho level (T1) when compared with higher tertiles of Klotho level (T2 and T3) in Fig. 10. These P-values after a log-rank test were less than 0.0001 and 0.0049 in the general and CKD populations, respectively. Furthermore, the relationships of Klotho with all-cause mortality in both categorical and continuous analyses in the Cox proportional hazards regression model were presented in Table 2 and Table 3. In the general population, using the T1 as the reference, the HR and 95% CI for all-cause mortality in the T2 and T3 were HR 0.648 (95% CI 0.529, 0.793) and HR 0.746 (95% CI 0.635, 0.877) in the unadjusted model (all P < 0.001), and the P for trend was 0.001. And in the adjusted model, the HR and 95% CI for all-cause mortality in the T2 was HR 0.741 (95% CI 0.598, 0.919) with P = 0.006. In the CKD population, using the T1 as the reference, the HR and 95% CI for all-cause mortality in the T3 was HR 0.685 (95% CI 0.506, 0.927) with P = 0.014 in the unadjusted model and the P for trend was 0.016. The continuous analysis showed that the effect of Klotho on all-cause mortality in the CKD population was HR 0.933 (95% CI 0.891, 0.976) with P = 0.003 in the unadjusted model and HR 0.942 (95% CI 0.890, 0.997) with P = 0.039 in the adjusted model.

**Table 2:** The association between Klotho and all-cause mortality in general population

Klotho	General population			
	Unadjusted model		Adjusted model	
	HR (95% CI)	P-value	HR (95% CI)	P-value
T1	Ref.			
T2	**0.648 (0.529, 0.793)**	** < 0.001**	**0.741 (0.598, 0.919)**	**0.006**
T3	**0.746 (0.635, 0.877)**	** < 0.001**	0.913 (0.757, 1.101)	0.340
P–t		**0.001**		0.385
Continuous	0.985 (0.959, 1.013)	0.291	1.009 (0.981, 1.037)	0.546

The unadjusted model was not adjusted by any covariates. The adjusted model was fully adjusted for sex, age, race, educational attainment, *BMI,* smoking status, *CVD,* DM and hypertension. *HR,* hazard ratio; *CI,* confidence interval; *P–t*, P for trend; *T1*, the lowest tertile of Klotho level; *T2*, the moderate tertile of Klotho level; *T3*, the highest tertile of Klotho level; *Ref,* reference

**Table 3:** The association between Klotho and all-cause mortality in CKD population

Klotho	CKD population			
	Unadjusted model		Adjusted model	
	HR (95% CI)	P-value	HR (95% CI)	P-value
T1	Ref.			
T2	0.827 (0.589, 1.161)	0.272	0.828 (0.601, 1.142)	0.249
T3	**0.685 (0.506, 0.927)**	**0.014**	0.711 (0.483, 1.048)	0.085
P–t		**0.016**		0.088
Continuous	**0.933 (0.891, 0.976)**	**0.003**	**0.942 (0.890, 0.997)**	**0.039**

The unadjusted model was not adjusted by any covariates. The adjusted model was fully adjusted for sex, age, race, educational attainment

*BMI,* smoking status, *CVD,* DM and hypertension. *HR,* hazard ratio; *CI,* confidence interval; *P–t,* P for trend; *T1*, the lowest tertile of Klotho level; *T2,* the moderate tertile of Klotho level; *T3,* the highest tertile of Klotho level; *Ref,* reference

## Discussion

Controversy exists regarding using Klotho as a potential biomarker in CKD [30]. A key contributing factor to this controversy is the absence of standardized techniques for assessing soluble Klotho [63]. Klotho expression in the kidney showed a strong parallel relationship with serum Klotho levels [64], so we utilized a combination of genetic and clinical data analysis to reduce the unreliable results from the inaccuracies of commercial enzyme-linked immunosorbent assays. Our study reconfirmed the good correlation between Klotho and renal function in CKD. In particular, we identified two major regulatory pathways through which Klotho exerted a protective role in CKD. Interestingly, we also found that there might be an optimal concentration range for Klotho to exert its biological functions.

Klotho is a transmembrane protein released into the bloodstream as soluble Klotho after being cleaved by metalloproteinases and exerts its multiple biological effects throughout the body via circulation [65]. Furthermore, the circulating Klotho is mainly produced in the kidney [11, 53], so we analyzed the expression of *Klotho* in the major organs/tissues of the body at the gene level and confirmed that *Klotho* was mainly expressed in the kidney. In addition, we found that *Klotho* was expressed primarily in the tubulointerstitium, especially in the distal convoluted tubules, which is consistent with a previous research report [1]. Specific deletion of the *Klotho* gene in mouse nephrons (mainly renal tubules) using Cre-Lox recombinant methods led to a significant decrease of about 80% in soluble Klotho levels in the serum [11], which confirmed that the primary source of Klotho in peripheral blood was the kidney, especially the renal tubules. Consequently, our research results showed that *Klotho* gene expression in the tubulointerstitium had a better correlation with the eGFR and BUN compared to *KL* expression in the glomerulus was well-founded, which guided us to focus on studying the main regulatory pathways of Klotho in the CKD tubulointerstitium.

Our analysis of snRNA-seq data from a normal adult kidney revealed that *Klotho* was also expressed in immune cells, which is consistent with a recent study report [66]. This study confirmed that Klotho was expressed in macrophages, monocytes and lymphocytes and exerted anti-inflammatory effects in these cells, which indirectly suggested that Klotho might be involved in regulating the inflammatory response in kidney disease. Interestingly, when we searched for the major negative regulatory pathway of Klotho in two large samples of CKD tubulointerstitial transcriptome datasets by *Klotho* gene functional enrichment analysis, we found that inflammatory response might be the main negative regulatory pathway of Klotho. Using GSVA analysis, we scored the inflammatory response gene sets in each sample from the two transcriptomic datasets above and found that the inflammatory response scores of each sample were significantly negatively correlated with *Klotho* gene expression, which further suggests that Klotho may negatively regulate the inflammatory response in the CKD tubulointerstitium. Given that CKD has persistent chronic low-grade inflammation [67], moreover, Klotho could inhibit NF-κB inflammatory pathway activation and monocyte inflammatory factor release [56], so we have further reason to believe that the main negative regulatory pathway through which Klotho exerts its protective effect in CKD is the inflammatory response. We also found significant immune cell infiltration in the tubulointerstitium of CKD by analysis of immune cell infiltration (22 immune cells), and the immune cells with upregulated percentage share were Mast cells resting, Macrophages M1, T cells gamma delta and B cells memory. Elevated numbers of Macrophages M1 and T cells gamma delta indicated inflammatory infiltration [68, 69], and elevated numbers of Mast cells resting and B cells memory indicated CKD in a chronic inflammatory state [70]. In particular, we also found that *Klotho* gene expression was significantly negatively correlated with the percentage of Neutrophils and T cells gamma delta cells and positively correlated with the percentage of T cells regulatory (Tregs). Moreover, our study also demonstrated that Klotho could affect monocyte infiltration in a CKD-like environment. These results further confirm that Klotho is involved in modulating the inflammatory response in CKD and may also be involved through T cells regulatory (Tregs) to regulate the immune response.

Surprisingly, when we searched for the major positive regulatory pathways of Klotho through *Klotho* gene function enrichment analysis in CKD tubulointerstitium, we found that lipid metabolism might be the leading positive regulatory pathway of Klotho, specifically the fatty acid β-oxidation pathway in the lipid metabolism pathway. To validate this finding, we performed GSVA analysis on lipid metabolism-related gene sets in samples from GSE104954 (CKD) and GSE108112 (CKD). Interestingly, we observed a significant positive correlation between the scores of the lipid metabolism-related gene sets and *Klotho* gene expression in each sample. This further supports our conclusion that Klotho may positively regulate lipid metabolism in CKD tubulointerstitium. We also found that the expression of two key nuclear transcription factors, *PPARA* and *PPARGC1A*, which regulated the expression of fatty acid oxidation-related enzymes, was significantly decreased in CKD tubulointerstitium. Moreover, the expression of fatty acid oxidation-related enzyme genes also significantly decreased, indicating that fatty acid oxidation was suppressed in CKD [57]. We also found that *KL* expression in CKD tubulointerstitium was dominantly positively correlated with the expression of *PPARA*, *PPARGC1A*, *CPT1A*, *ACOX1* and *ACOX2*. Moreover, klotho could positively regulate the expression of the above genes in vitro experiments. In particular, Klotho could positively regulate the expression of PPARα and PGC1α. Considering that one previous study has reported that Klotho could improve hepatic lipid metabolism abnormalities in type 2 diabetic mice by directly interacting with the type 1 insulin-like growth factor receptor (IGF1R) to upregulate *PPARA* expression [14], we have more reasons to believe that Klotho positively regulates the lipid metabolism in CKD. A recent study reported that Klotho was involved in the positive regulation of lipid metabolism in renal tubular epithelial cells in acute kidney injury [71]. Furthermore, our in vitro experiments also confirmed that Klotho positively regulated lipid metabolism in HK-2 exposed to a CKD-like environment. Thus, Klotho is essential for maintaining lipid homeostasis in renal tubular epithelial cells.

To verify whether Klotho can regulate inflammatory response and lipid metabolism in CKD, we established an in vitro experimental model to simulate the CKD environment. Our study confirmed that TGF-β1 and palmitic acid (TPA) induced inflammatory response and abnormal lipid metabolism in HK-2. Moreover, TPA significantly decreased the expression of Klotho and caused abnormal changes in mitochondrial morphology, but the addition of rKlotho improved mitochondrial morphology. These results suggest that Klotho may regulate mitochondrial function. A study confirmed that Klotho protected against H2O2-induced human periodontal stem cell injury by regulating mitochondrial function [45]. Given that Klotho can regulate mitochondrial function, this implies that Klotho may also regulate fatty acid β-oxidation in mitochondrion. Our study confirmed that Klotho regulated fatty acid β-oxidation by modulating the expression of mitochondrial fatty acid oxidation-related enzymes. Targeting Smad3 can improve fatty acid oxidation and inflammatory response [57, 60], and our experiments have shown that Klotho can inhibit TPA-induced phosphorylation of Smad3, increase the expression of *PPARGC1A*, and suppress the release of inflammatory factors. These results indirectly demonstrate that Klotho can improve fatty acid oxidation and alleviate inflammatory response partially by inhibiting the TGF-β1/Smad3 signaling pathway. Our experiments also confirmed that Klotho positively regulated the expression of *PPARA*, another key nuclear transcription factor of fatty acid oxidation [72], so Klotho can indirectly improve fatty acid oxidation by positively regulating the expression of *PPARA* and *PPARGC1A*. Interestingly, the agonist of *PPARA* (WY-14643) exerts anti-inflammatory effects through *PPARA* [73], and our experiments confirmed that Klotho up-regulated the expression of *PPARA*, which indirectly demonstrates that Klotho can also exert anti-inflammatory effects through *PPARA*. Klotho also regulates the expression of adipogenic and fatty acid transporter genes [14], as confirmed by our experiments, but further experiments are needed to investigate the exact mechanism of regulation. Klotho has been shown to modulate the inflammatory response in previous studies [74, 75], and our study validated this view and emphasized its predominant role in CKD.

The landmark study by Kang et al. identified inflammation and metabolism as the primary dysregulated pathways in CKD tubulointerstitium. Also, it showed that metabolic abnormalities primarily encompass fatty acid oxidation dysregulation [57]. Based on our findings that Klotho may predominantly negatively regulate inflammatory response and positively regulate lipid metabolism (especially fatty acid β-oxidation) in CKD tubulointerstitium, it is reasonable to suggest that Klotho may protect renal function in CKD patients by regulating inflammatory response and lipid metabolism. To verify this speculation, we included 10 years of NHANES clinical data containing the Klotho indicator to conduct the study. Considering that Klotho could be expressed in immune cells and modulate their anti-inflammatory effects [66], as well as the ease of obtaining whole blood cell test results, we used WBC, Neu, Lym, Mono, SII, PIV, SIRI, NLR, MLR and PLR as inflammatory biomarkers. Our results found that all inflammatory biomarkers except Lym and PLR were significantly elevated in the CKD population, which validated the presence of persistent systemic inflammation in CKD patients [76]. We also found that Klotho decreased considerably in the CKD population (Table 1), which is consistent with our bioinformatics analysis and a previous study [21]. Proinflammatory cytokines could influence Klotho expression, and Klotho could also act as an anti-inflammatory modulator [77], so what was the relationship between Klotho and the inflammatory response in the CKD population? By generalized linear regression analysis, we found that Klotho was negatively associated with most inflammatory biomarkers in the general and CKD populations. Suppressing inflammation could improve kidney function [78], which makes us more confident that Klotho can improve renal function by mediating the inflammatory response. We then confirmed that Klotho could improve renal function by regulating inflammatory biomarkers in both the general population and the CKD population through mediation analysis, suggesting that negative regulation of inflammatory response by Klotho has a critical position in protecting renal function.

There are also significant lipid metabolism disorders in CKD [79], and our study found that TC and TG were all significantly elevated in the CKD population, which confirmed that CKD patients had apparent lipid metabolism disorders. Considering our finding that Klotho is mainly positively associated with lipid metabolism in CKD tubulointerstitium, we also studied the relationship between Klotho and lipids in the peripheral blood of CKD patients. We found that Klotho was negatively correlated with TC and TG in the general population and with TG in the CKD population. Only the relationship between Klotho and TG was demonstrated in the CKD population, which might be related to the small size of the CKD population, or it might be related to the better linear relationship between TG and CKD progression than TC [58]. These speculations need to be confirmed by a larger sample of the CKD population. We also found that Klotho might regulate serum urea nitrogen through TG, and uric acid through TC and TG in the general population by mediation analysis, but there was no significant mediation effect in the CKD population, which might be related to the small size of the CKD population. Since biomarkers of inflammation may mediate the association between Klotho and renal function in the CKD population in our study, this implies that the effects of Klotho regulation of inflammatory response on renal function may be more pronounced than the effects of Klotho regulation of lipid metabolism on renal function. The regulation of lipid metabolism by Klotho that affects renal function may require an intermediate step of inflammatory response. Therefore, we need to increase the sample size for research.

The blood lipid abnormalities in most CKD patients without overt proteinuria are usually characterized by hypertriglyceridemia [79]. Our results confirmed these findings. In our study, CKD participants did not have significant proteinuria but had blood lipid abnormalities, mainly with elevated triglyceride levels (Table 1). Triglyceride was remarkably elevated and negatively associated with Klotho in the CKD population, which may be related to the inhibition of Klotho regulation of fatty acid oxidation. Based on the fact that Klotho was negatively associated with inflammatory response and positively associated with lipid metabolism and renal function in the general and CKD populations, we believed that Klotho could reduce the risk of all-cause mortality in general and CKD populations. Our views were confirmed by survival analysis and the Cox proportional hazards regression model, and high levels of Klotho could reduce the risk of all-cause mortality in general and CKD populations [80, 81]. Interestingly, when we analyzed the relationship between Klotho concentration and inflammatory biomarkers, lipid biomarkers and renal function biomarkers, we found that Klotho had a threshold effect in the same concentration range, suggesting that there was an optimal concentration range (around 1000 pg/ml) for Klotho to exert its biological function. A recent study found that all-cause mortality was lowest in the general population at Klotho concentrations around 1000 pg/ml (961.4 to 1140.0 pg/ml) [82]. These findings provided a basis for clinically monitoring Klotho concentration, but the precise concentration range required further investigation. We also found that in NHANES clinical data, both in the general population and in the CKD population, both in the unadjusted model and in the adjusted model, Klotho correlated well with biomarkers of renal function, except for UACR. Combined with the results of our bioinformatics analysis that *Klotho* gene expression is well associated with renal function, we had to conclude that Klotho could be a biomarker for CKD.

The limitations of this study are as follows. Firstly, our study is a bioinformatics analysis and combined observational survey study, but multilevel validation methods and mediation analysis are used in the study to improve the reliability of the evidence. Secondly, the mechanistic pathways related to the negative regulation of inflammatory response and positive regulation of lipid metabolism by Klotho in CKD have been explored partly. For example, Klotho could regulate Smad3 phosphorylation and *PPARA* expression to regulate both inflammatory response and lipid metabolism, but other regulatory mechanisms need to be uncovered. In addition, the FGF-23/Klotho signaling axis plays a vital role in maintaining phosphorus balance and regulating vitamin D metabolism in CKD. Our study showed that the expression of *FGF23* in the renal tubulointerstitium was significantly decreased and was not significantly associated with the expression of *KL* (Additional file 2: Fig. S3A-C). This is because the main source of FGF23 is not the kidney but bone tissue. Due to the lack of serum FGF23 in our clinical data, our study did not reflect the relationship between FGF23 and Klotho well. Finally, the number of people with CKD included in the NHANES study is still not large enough, and a study with a larger sample size of the CKD population is needed to validate our findings.

## Conclusions

Due to the current lack of standardized techniques for detecting soluble Klotho, research on Klotho as a diagnostic or prognostic marker for CKD is limited. Our study reconfirmed a good correlation between Klotho and renal function through combined genetic and clinical data analysis in CKD. We also found an optimal concentration range for Klotho to effectively exert its biological function in vivo. Additionally, we found that inflammatory response and lipid metabolism might be the primary regulatory pathways of Klotho in CKD tubulointerstitium. We demonstrated that Klotho could reduce cellular inflammatory response and improve cellular lipid metabolism by establishing an in vitro model similar to CKD. We used NHANES clinical data to indirectly confirm that Klotho could improve renal function by regulating inflammatory response and lipid metabolism and could also reduce the risk of all-cause mortality in CKD patients. The above results suggest that Klotho may primarily exert protective effects in the kidney by regulating inflammatory response and lipid metabolism in CKD, and it can serve as a potential biomarker for CKD.

### Supplementary Information

**Additional file 1: Table S1.** PCR primer design. **Table S2.** The association of Klotho with inflammatory biomarkers, lipid biomarkers and renal function in general population. **Table S3.** The association of Klotho with inflammatory biomarkers, lipid biomarkers and renal function in CKD population. **Table S4.** Mediation effects of inflammation and lipid biomarkers on the association of Klotho with renal function in general population. **Table S5.** Mediation effects of inflammation and lipid biomarkers on the association of Klotho with renal function in CKD population.

**Additional file 2: Fig. S1.** The effect of Klotho on monocyte invasion. **Fig. S2.** Klotho improved PPARα and PGC1α expression in HK-2 treated with TPA. **Fig. S3.** Correlation analysis of *Klotho* gene expression with *FGF23* expression in CKD tubulointerstitium.

**Additional file 3:** Supplementary figure legends.**Fig. S1.** The effect of Klotho on monocyte invasion. **A** Analysis of monocyte invasion numbers in the transwell invasion assay. The assay was induced with RPMI1640 medium containing fetal bovine serum (FBS). **B** Analysis of monocyte invasion numbers in the transwell invasion assay. The assay was induced with conditioned medium. The conditioned medium obtained from HK-2 with normal treatment, TPA treatment or TPA + rKlotho treatment for 48 h. Scale bars = 100 μm. FBS-, FBS-free RPMI1640 medium; FBS+, 10% FBS RPMI1640 medium. The results were mean ± SEM of three independent experiments. **** P < 0.0001. **Fig. S2.**Klotho improved PPARα and PGC1α expression in HK-2 treated with TPA. **A-C** Western blot analysis of PPARα and PGC1α expression in HK-2 treated with TPA and TPA + rKlotho. The molecular weights of PPARα and PGC1α were 52 and 91 kDa, respectively. The results were mean ± SEM of three independent experiments. * P < 0.05, ** P < 0.01. **Fig. S3. **Correlation analysis of Klotho gene expression with FGF23 expression in CKD tubulointerstitium. A Comparative analysis of FGF23 expression in tubulointerstitium of Living Donors (n = 21) and CKD patients (n = 169) based on the Mann-Whitney test in GSE104954. **B**, **C** The Spearman correlation analysis of *KL* expression with *FGF23* expression in GSE104954 (CKD) and GSE108112 (CKD). R represented correlation coefficient. *** P < 0.001.

## Data Availability

The GTEx RNA-seq data of *KL* are available from the Human Protein Atlas (HPA) website under the label “tissue” (https://www.proteinatlas.org/ENSG00000133116-KL/tissue). The Ju CKD Glom, Ju CKD TubInt, Sampson Nephrotic Syndrome TubInt and Woroniecka Diabetes TubInt datasets are attainable from the Nephroseq v5 database (http://v5.nephroseq.org/ or www.nephroseq.org). GSE21785, GSE118184, GSE104954 and GSE108112 datasets are downloaded from the Gene Expression Omnibus (GEO) database (https://www.ncbi.nlm.nih.gov/geo/). The observational survey data on the overall health and nutritional status of the noninstitutionalized population across the United States can be downloaded for free from the National Health and Nutrition Examination Survey (NHANES) website under the label “Survey Data and Documentation” (https://wwwn.cdc.gov/nchs/nhanes/Default.aspx).

## Abbreviations

CKD: Chronic kidney disease
NHANES: National Health and Nutrition Examination Survey
GLM: Generalized linear model
RCS: Restricted cubic spline
eGFR: Estimated glomerular filtration rate
RNA-seq: RNA-sequencing
snRNA-seq: Single nucleus RNA sequencing
DAVID: Database for annotation, visualization, and integrated discovery
GEO: Gene Expression Omnibus
GO: Gene ontology
KEGG: Kyoto Encyclopedia of Genes and Genomes
GSVA: Gene set variation analysis
NCHS: National Center for Health Statistics
WBC: White blood cell
Neu: Neutrophil
Lym: Lymphocyte
Mono: Monocyte
SII: Systemic immune-inflammation index
PIV: Pan-immune-inflammation value
SIRI: Systemic inflammatory response index
SCI: Systemic chronic inflammation
NLR: Neutrophil-to-lymphocyte ratio
MLR: Monocyte-to-lymphocyte ratio
PLR: Platelet-to-lymphocyte ratio
HDL: High density lipoprotein
LDL: Low density lipoprotein
TC: Total cholesterol
TG: Triglyceride
UACR: Urine albumin-to-creatinine ratio
DM: Diabetes mellitus
CVD: Cardiovascular disease
BUN: Blood urea nitrogen
PPARA: Peroxisome proliferator-activated receptor α
PPARGC1A: PPAR-γ coactivator-1α
BMI: Body mass index
95% CI: 95% Confidence interval
Ref: Reference
HR: Hazard ratio
TPA: TGF-β1 and palmitic acid
PA: Palmitic acid
rKlotho: Recombinant human Klotho
