# Correction to: Klotho exerts protection in chronic kidney disease associated with regulating inflammatory response and lipid metabolism

**DOI:** 10.1186/s13578-024-01263-z

**Published:** 2024-07-26

**Authors:** Junhui Liu, Huaicheng Wang, Qinyu Liu, Shushu Long, Yanfang Wu, Nengying Wang, Wei Lin, Gang Chen, Miao Lin, Junping Wen

**Affiliations:** 1 Shengli Clinical Medical College of Fujian Medical University, Fujian Medical University, Fuzhou, China; 2 Department of Endocrinology, Fujian Provincial Hospital, Shengli Clinical Medical College of Fujian Medical University, Fuzhou, China; 3 Department of Nephrology, Provincial Clinical College, Fujian Provincial Hospital, Fujian Medical University, Fuzhou, China

**Correction to: Cell Biosci (2024) 14: 46**

10.1186/s13578-024-01226-4

In the publication of this article [1], the authors have identified two errors in the initial version. Now they have been included in this correction.

Firstly, for the calculation of the Urine Albumin-to-Creatinine Ratio (UACR), the authors mistakenly used incorrect parameters when calculating due to the variable mislabeling in data processing. Eventually, the wrong calculation of UACR led to its incorrect values in the baseline characteristics of NHANES participants (Table 1) and the corresponding results in the generalized linear model (Additional file 1: Tables S2, S3) and mediation analysis (Additional file 1: Tables S4, S5). As a result of this error, the following changes have been made to the originally published version of this article:

The fourth and fifth sentences of the eleventh paragraph of the Results originally read “Overall, there were statistically significant differences between non-CKD and CKD participants in terms of sex, age, race, educational attainment, BMI, smoking status, CVD, DM, hypertension, Klotho, TC, TG, WBC, Neu, Mono, SII, NLR, MLR, SIRI, PIV, eGFR, serum creatinine, uric acid and serum urea nitrogen (all P < 0.05). However, no significant differences were observed between the two groups for other variables, including HDL, LDL, Lym, PLR and UACR (all P > 0.05).” In the corrected version, UACR has a statistically significant difference between non-CKD and CKD participants. The sentences are corrected as “Overall, there were statistically significant differences between non-CKD and CKD participants in terms of sex, age, race, educational attainment, BMI, smoking status, CVD, DM, hypertension, Klotho, TC, TG, WBC, Neu, Mono, SII, NLR, MLR, SIRI, PIV, eGFR, serum creatinine, UACR, uric acid and serum urea nitrogen (all P < 0.05). However, no significant differences were observed between the two groups for other variables, including HDL, LDL, Lym and PLR (all P > 0.05).”

The second and third sentences of the twelfth paragraph of the Results originally read “Using the lowest tertile of Klotho level (T1) as the reference, the β and 95% CI for TC, eGFR, serum urea nitrogen, serum creatinine, and uric acid in the moderate tertile of Klotho level (T2) were β -3.227 (95% CI -6.288, -0.167), β 1.689 (95% CI 0.596, 2.782), β -0.409 (95% CI -0.668, -0.150), β -0.033 (95% CI -0.050, -0.016) and β -0.172 (95% CI -0.255, -0.088) in the adjusted model (all P < 0.05). Furthermore, using the T1 as the reference, the β and 95% CI for WBC, Neu, PLR, PIV, SII, TC, TG, eGFR, serum urea nitrogen, serum creatinine and uric acid in the highest tertile of Klotho level (T3) were β -0.174 (95% CI -0.319, -0.028), β -0.160 (95% CI -0.288, -0.032), β -5.865 (95% CI -9.028, -2.702), β -27.806 (95% CI -43.335, -12.277), β -43.053 (95% CI -65.437, -20.669), β -4.335 (95% CI -7.629, -1.041), β -12.756 (95% CI -23.960, -1.552), β 2.799 (95% CI 1.792, 3.806), β -0.589 (95% CI -0.892, -0.285), β -0.050 (95% CI -0.068, -0.032) and β -0.351 (95% CI -0.434, -0.269) in the adjusted model (all P < 0.05).” The sentences are corrected as “Using the lowest tertile of Klotho level (T1) as the reference, the β and 95% CI for TC, eGFR, serum urea nitrogen, serum creatinine, uric acid and UACR in the moderate tertile of Klotho level (T2) were β -3.227 (95% CI -6.288, -0.167), β 1.689 (95% CI 0.596, 2.782), β -0.409 (95% CI -0.668, -0.150), β -0.033 (95% CI -0.050, -0.016), β -0.172 (95% CI -0.255, -0.088) and β -13.721 (95% CI -25.918, -1.525) in the adjusted model (all P < 0.05). Furthermore, using the T1 as the reference, the β and 95% CI for WBC, Neu, PLR, PIV, SII, TC, TG, eGFR, serum urea nitrogen, serum creatinine, uric acid and UACR in the highest tertile of Klotho level (T3) were β -0.174 (95% CI -0.319, -0.028), β -0.160 (95% CI -0.288, -0.032), β -5.865 (95% CI -9.028, -2.702), β -27.806 (95% CI -43.335, -12.277), β -43.053 (95% CI -65.437, -20.669), β -4.335 (95% CI -7.629, -1.041), β -12.756 (95% CI -23.960, -1.552), β 2.799 (95% CI 1.792, 3.806), β -0.589 (95% CI -0.892, -0.285), β -0.050 (95% CI -0.068, -0.032), β -0.351 (95% CI -0.434, -0.269) and β -14.360 (95% CI -26.809, -1.912) in the adjusted model (all P < 0.05).”

Replace the incorrect UACR calculation results in Table 1 and Additional file 1: Tables S2, S3, S4, S5. The correct Additional file 1: Tables S2, S3, S4, S5 are available on the Cell & Bioscience’s website from the date of publication of this note.

Secondly, for the presentation of continuous variables of the baseline characteristics of NHANES participants (Table 1), the authors mistakenly labeled “SE” (standard error) as “SD” (standard deviation).

As a result of this error, the following changes have been made to the originally published version of this article:

The eighth sentence of the nineteenth paragraph of the Methods originally read “Continuous variables were described as mean ± standard deviation (SD), while categorical variables were presented as n (%).” In the corrected version, the sentence is corrected as “Continuous variables were described as mean ± standard error (SE), while categorical variables were presented as n (%).” The same correction is made to the Table 1 legend (“Continuous variables were presented as Mean ± SD” is replaced by “Continuous variables were presented as Mean (SE)”).

The correct Table 1 is given in this correction.

**Table 1:** Descriptive characteristics of NHANES participants

Characteristics	General population	Population without CKD	Population with CKD	*P*-value
Sex				**0.003**
Male	4543 (46.74)	3870 (46.35)	673 (39.91)	
Female	5176 (53.26)	4329 (53.65)	847 (60.09)	
Age in years	53.96 (0.16)	53.11 (0.17)	59.81 (0.33)	**< 0.001**
Age				**< 0.001**
40–50	3445 (35.45)	3139 (41.58)	306 (22.25)	
50–60	2799 (28.8)	2453 (33.80)	346 (25.63)	
60–70	2341 (24.09)	1871 (18.02)	470 (29.14)	
> = 70	1134 (11.67)	736 (6.60)	398 (22.97)	
Race				**< 0.001**
Mexican American	1643 (16.91)	1380 (7.29)	263 (8.09)	
Non-Hispanic White	4002 (41.18)	3400 (71.88)	602 (67.58)	
Non-Hispanic Black	1925 (19.81)	1559 (9.01)	366 (12.62)	
Other Races	2149 (22.11)	1860 (11.83)	289 (11.71)	
Educational attainment				**< 0.001**
< 11th grade	2683 (27.61)	2177 (15.44)	506 (21.18)	
High school graduate	2135 (21.97)	1785 (21.71)	350 (23.65)	
Some college or AA degree	2653 (27.3)	2253 (30.48)	400 (30.05)	
College graduate or above	2248 (23.13)	1984 (32.37)	264 (25.13)	
BMI				**< 0.001**
Normal	2595 (26.7)	2258 (28.33)	337 (22.65)	
Overweight	3382 (34.8)	2913 (35.92)	469 (31.21)	
Obese	3742 (38.5)	3028 (35.75)	714 (46.13)	
Smoking status				**0.001**
Never	5182 (53.32)	4436 (54.41)	746 (48.26)	
Former	2485 (25.57)	2059 (26.21)	426 (31.13)	
Current	2052 (21.11)	1704 (19.38)	348 (20.60)	
CVD				**< 0.001**
Yes	721 (7.42)	472 (4.44)	249 (12.87)	
No	8998 (92.58)	7727 (95.56)	1271 (87.13)	
DM				**< 0.001**
Yes	1670 (17.18)	1131 (9.87)	539 (28.48)	
No	8049 (82.82)	7068 (90.13)	981 (71.52)	
Hypertension				**< 0.001**
Yes	4386 (45.13)	3343 (36.94)	1043 (63.34)	
No	5333 (54.87)	4856 (63.06)	477 (36.66)	
Klotho (pg/ml)	859.53 (5.97)	863.18 (6.36)	834.54 (9.88)	**0.008**
Biomarkers of lipid				
HDL (mg/dl)	55.33 (0.35)	55.42 (0.34)	54.70 (1.01)	0.464
LDL (mg/dl)	126.38 (0.74)	126.77 (0.75)	123.52 (1.92)	0.094
TC (mg/dl)	209.94 (0.75)	209.55 (0.75)	212.68 (1.63)	**0.047**
TG (mg/dl)	162.13 (2.16)	159.07 (2.34)	183.05 (4.67)	**< 0.001**
Biomarkers of inflammation				
WBC (1000 cells/ul)	6.57 (0.06)	6.51 (0.06)	7.01 (0.11)	**< 0.001**
Neu (1000 cells/ul)	3.87 (0.04)	3.82 (0.04)	4.27 (0.09)	**< 0.001**
Lym (1000 cells/ul)	1.92 (0.02)	1.92 (0.02)	1.92 (0.05)	0.966
Mono (1000 cells/ul)	0.53 (0.01)	0.52 (0.01)	0.56 (0.01)	**0.004**
SII	527.75 (8.13)	517.70 (8.20)	601.61 (19.63)	**< 0.001**
NLR	2.17 (0.03)	2.12 (0.03)	2.49 (0.07)	**< 0.001**
MLR	0.29 (0.00)	0.29 (0.00)	0.32 (0.01)	**< 0.001**
PLR	136.50 (1.39)	136.20 (1.45)	138.76 (2.28)	0.269
SIRI	1.17 (0.02)	1.14 (0.02)	1.43 (0.05)	**< 0.001**
PIV	288.06 (5.47)	279.62 (5.64)	350.07 (15.86)	**< 0.001**
Biomarkers of renal function				
eGFR (mL/min/1.73m^2^)	89.44 (0.35)	91.59 (0.31)	74.72 (0.90)	**< 0.001**
Serum creatinine (mg/dl)	0.87 (0.00)	0.84 (0.00)	1.08 (0.02)	**< 0.001**
UACR (mg/g)	27.77 (2.41)	7.89 (0.09)	164.85 (18.52)	**< 0.001**
Uric acid (mg/dl)	5.40 (0.03)	5.33 (0.03)	5.94 (0.06)	**< 0.001**
Serum urea nitrogen (mg/dl)	13.58 (0.10)	13.19 (0.09)	16.25 (0.25)	**< 0.001**

CKD, chronic kidney disease; BMI, body mass index; CVD, cardiovascular disease; DM, diabetes mellitus; HDL, high density lipoprotein; LDL, low density lipoprotein; TC, total cholesterol; TG, triglyceride; WBC, white blood cell; Neu, neutrophil; Lym, lymphocyte; Mono, monocyte; SII, systemic immune-inflammation index; NLR, neutrophil to lymphocyte ratio; MLR, monocyte to lymphocyte ratio; PLR, platelet to lymphocyte ratio; SIRI, systemic inflammatory response index; PIV, pan-immune-inflammation value; eGFR, estimated glomerular filtration rate; UACR, Urine Albumin-to-Creatinine Ratio. Continuous variables were presented as Mean (SE), and categorical variables were presented as N (%)

### Electronic supplementary material

Below is the link to the electronic supplementary material.

Supplementary Material 1

Supplementary Material 2

Supplementary Material 3

Supplementary Material 4

## References

[1] Liu J, Wang H, Liu Q, Long S, Wu Y, Wang N, et al. Klotho exerts protection in chronic kidney disease associated with regulating inflammatory response and lipid metabolism. Cell Biosci. 2024;14(1):46. 10.1186/s13578-024-01226-4. 38584258 10.1186/s13578-024-01226-4 PMC11000353

