# Supplementary figures and images for: Klotho exerts protection in chronic kidney disease associated with regulating inflammatory response and lipid metabolism
Source: Cell Biosci. 2024 Apr 7;14:46. doi: 10.1186/s13578-024-01226-4 (PMC11000353; doi:10.1186/s13578-024-01226-4)

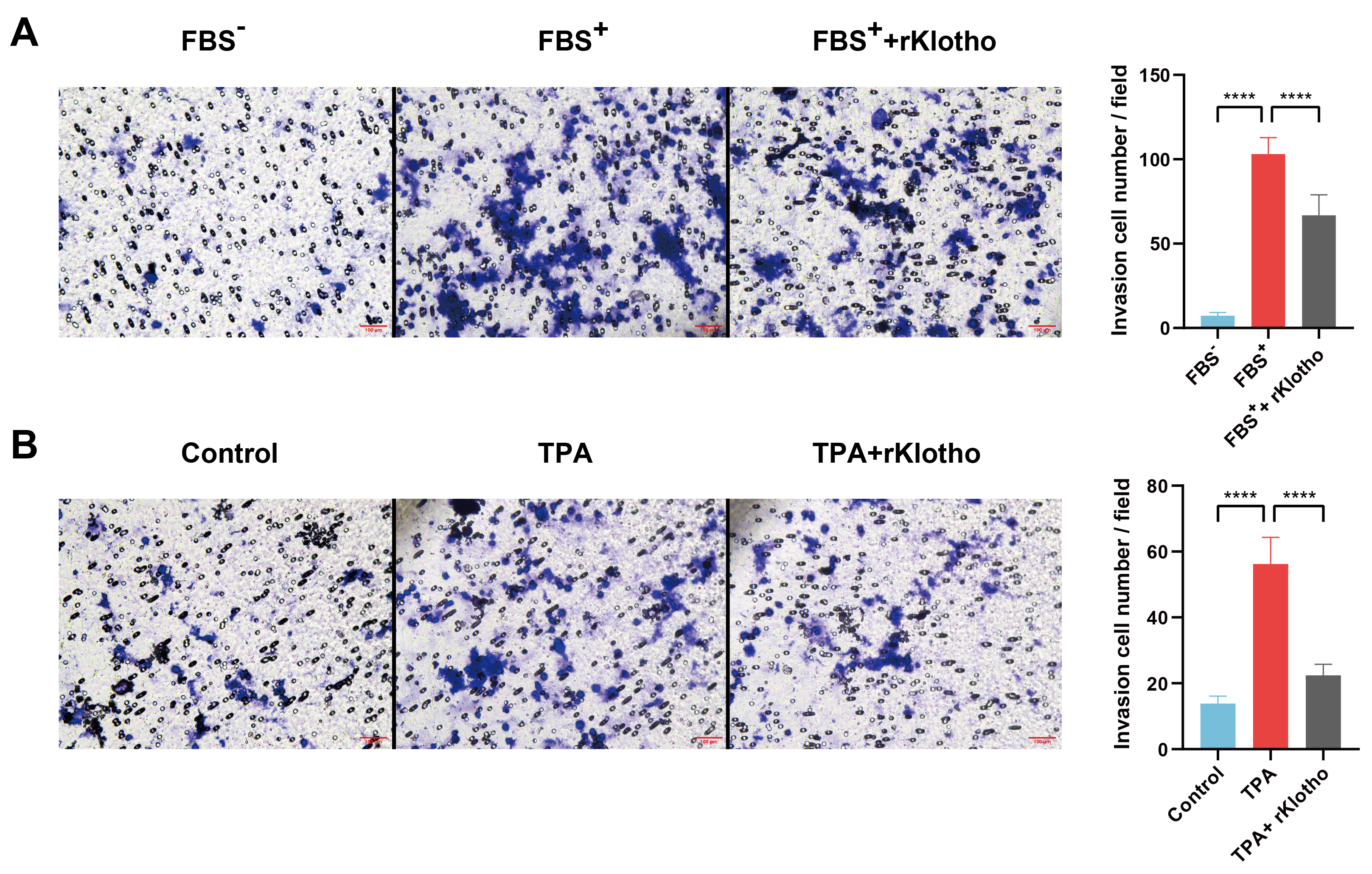

Supplement: Supplementary file 2 — Additional file 2: Fig. S1. The effect of Klotho on monocyte invasion. Fig. S2. Klotho improved PPARα and PGC1α expression in HK-2 treated with TPA. Fig. S3. Correlation analysis of Klotho gene expression with FGF23 expression in CKD tubulointerstitium. [file 13578_2024_1226_MOESM2_ESM.zip › Additional file 1/Fig. S1.tif]

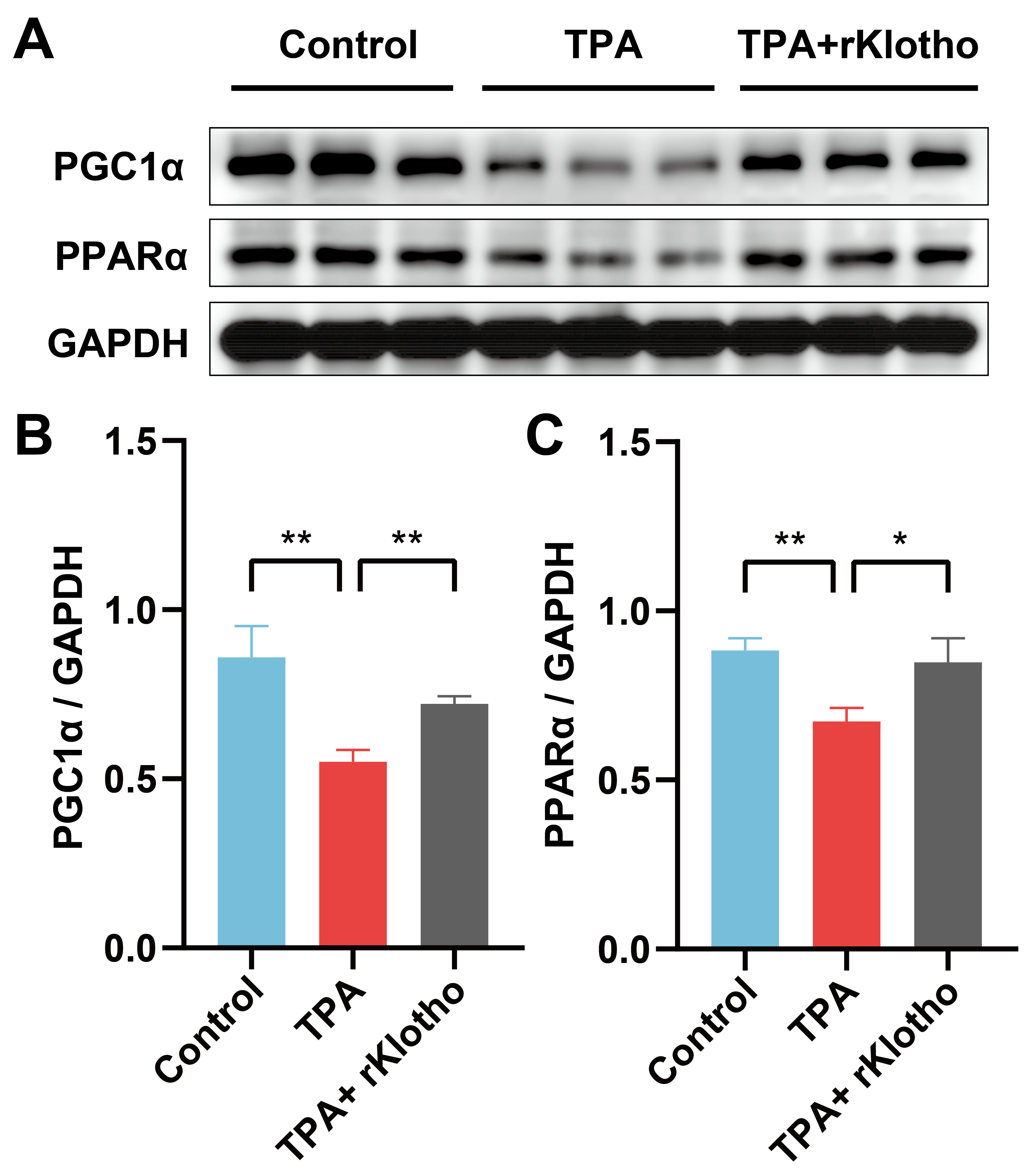

Supplement: Supplementary file 2 — Additional file 2: Fig. S1. The effect of Klotho on monocyte invasion. Fig. S2. Klotho improved PPARα and PGC1α expression in HK-2 treated with TPA. Fig. S3. Correlation analysis of Klotho gene expression with FGF23 expression in CKD tubulointerstitium. [file 13578_2024_1226_MOESM2_ESM.zip › Additional file 1/Fig. S2.tif]

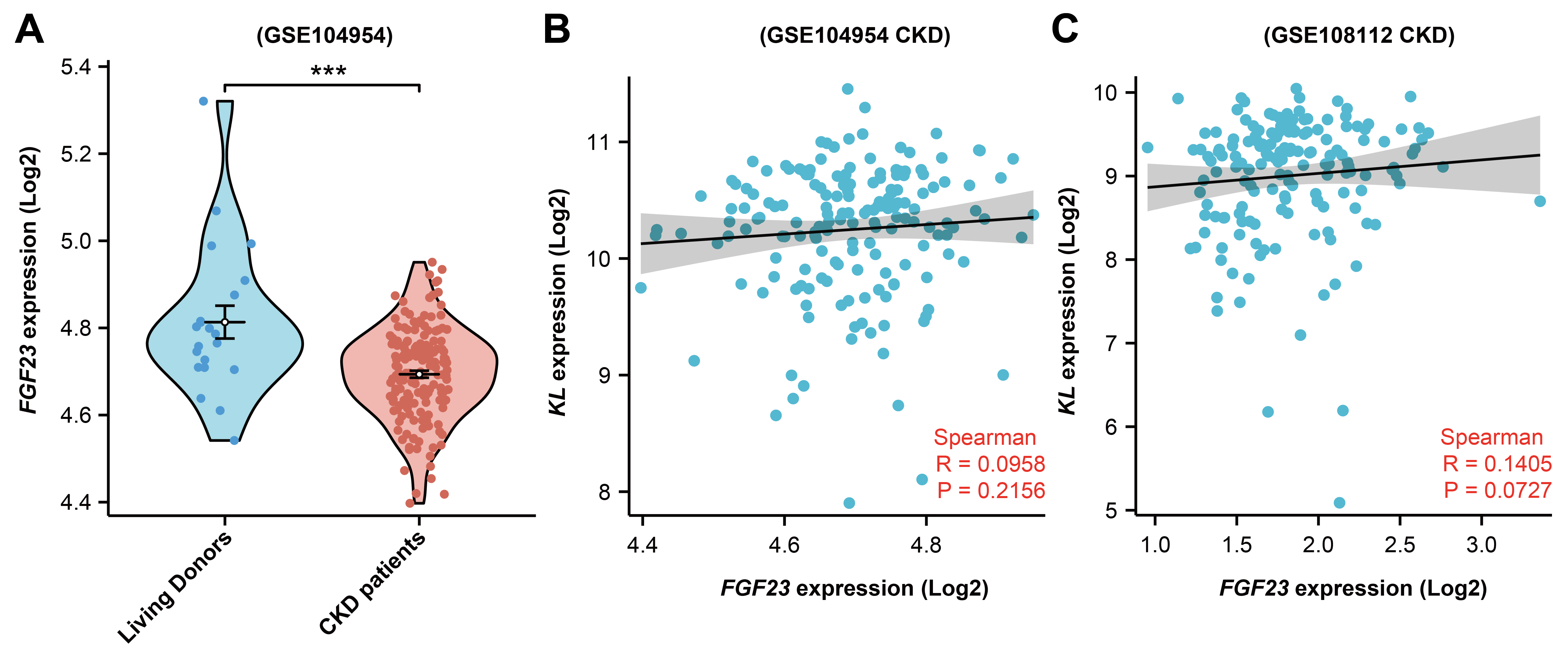

Supplement: Supplementary file 2 — Additional file 2: Fig. S1. The effect of Klotho on monocyte invasion. Fig. S2. Klotho improved PPARα and PGC1α expression in HK-2 treated with TPA. Fig. S3. Correlation analysis of Klotho gene expression with FGF23 expression in CKD tubulointerstitium. [file 13578_2024_1226_MOESM2_ESM.zip › Additional file 1/Fig. S3.tif]
