# Supplementary material for: Klotho exerts protection in chronic kidney disease associated with regulating inflammatory response and lipid metabolism
Source: Cell Biosci. 2024 Apr 7;14:46. doi: 10.1186/s13578-024-01226-4 (PMC11000353; doi:10.1186/s13578-024-01226-4)
Supplement: Supplementary file 3 — Additional file 3: Supplementary figure legends.Fig. S1. The effect of Klotho on monocyte invasion. A Analysis of monocyte invasion numbers in the transwell invasion assay. The assay was induced with RPMI1640 medium containing fetal bovine serum (FBS). B Analysis of monocyte invasion numbers in the transwell invasion assay. The assay was induced with conditioned medium. The conditioned medium obtained from HK-2 with normal treatment, TPA treatment or TPA + rKlotho treatment for 48 h. Scale bars = 100 μm. FBS-, FBS-free RPMI1640 medium; FBS+, 10% FBS RPMI1640 medium. The results were mean ± SEM of three independent experiments. **** P < 0.0001. Fig. S2.Klotho improved PPARα and PGC1α expression in HK-2 treated with TPA. A-C Western blot analysis of PPARα and PGC1α expression in HK-2 treated with TPA and TPA + rKlotho. The molecular weights of PPARα and PGC1α were 52 and 91 kDa, respectively. The results were mean ± SEM of three independent experiments. * P < 0.05, ** P < 0.01. Fig. S3. Correlation analysis of Klotho gene expression with FGF23 expression in CKD tubulointerstitium. A Comparative analysis of FGF23 expression in tubulointerstitium of Living Donors (n = 21) and CKD patients (n = 169) based on the Mann-Whitney test in GSE104954. B, C The Spearman correlation analysis of KL expression with FGF23 expression in GSE104954 (CKD) and GSE108112 (CKD). R represented correlation coefficient. *** P < 0.001. [file 13578_2024_1226_MOESM3_ESM.docx]

**Supplementary figure legends**

**Fig. S1** The effect of Klotho on monocyte invasion. **A** Analysis of monocyte invasion numbers in the transwell invasion assay. The assay was induced with RPMI1640 medium containing fetal bovine serum (FBS). **B** Analysis of monocyte invasion numbers in the transwell invasion assay. The assay was induced with conditioned medium. The conditioned medium obtained from HK-2 with normal treatment, TPA treatment or TPA + rKlotho treatment for 48 h. Scale bars = 100 μm. FBS^-^, FBS-free RPMI1640 medium; FBS**^+^**, 10% FBS RPMI1640 medium. The results were mean ± SEM of three independent experiments. **** P < 0.0001.

**Fig. S2** Klotho improved PPARα and PGC1α expression in HK-2 treated with TPA. **A**-**C** Western blot analysis of PPARα and PGC1α expression in HK-2 treated with TPA and TPA + rKlotho. The molecular weights of PPARα and PGC1α were 52 and 91 kDa, respectively. The results were mean ± SEM of three independent experiments. * P < 0.05, ** P < 0.01.

**Fig. S3** Correlation analysis of *Klotho* gene expression with *FGF23* expression in CKD tubulointerstitium. **A** Comparative analysis of *FGF23* expression in tubulointerstitium of Living Donors (n = 21) and CKD patients (n = 169) based on the Mann-Whitney test in GSE104954. **B**, **C** The Spearman correlation analysis of *KL* expression with *FGF23* expression in GSE104954 (CKD) and GSE108112 (CKD). R represented correlation coefficient. *** P < 0.001.
