# Supplementary material for: Correction to: Klotho exerts protection in chronic kidney disease associated with regulating inflammatory response and lipid metabolism
Source: Cell Biosci. 2024 Jul 26;14:97. doi: 10.1186/s13578-024-01263-z (PMC11282863; doi:10.1186/s13578-024-01263-z)
Supplement: Supplementary file 2 — Supplementary Material 2 [file 13578_2024_1263_MOESM2_ESM.docx]

**Table S3** The association of Klotho with inflammatory biomarkers, lipid biomarkers and renal function in CKD population.

| **Biomarkers** | **Serum soluble Klotho** | | | | | | | | | | | | |
| --- | --- | --- | --- | --- | --- | --- | --- | --- | --- | --- | --- | --- | --- |
|  |  | **Unadjusted model** | | | | | | **Adjusted model** | | | | | |
|  | T1 | T2 | | T3 | | Continuous | | T2 | | T3 | | Continuous | |
|  | β | β (95% CI) | P-value | β (95% CI) | P-value | β (95% CI) | P-value | β (95% CI) | P-value | β (95% CI) | P-value | β (95% CI) | P-value |
| **Biomarkers of inflammation** (n = 1,514) |  |  |  |  |  |  |  |  |  |  |  |  |  |
| WBC | Ref. | -0.362 (-0.690, -0.034) | **0.031** | -0.492 (-0.797, -0.188) | **0.002** | -0.068 (-0.115, -0.020) | **0.006** | -0.365 (-0.665, -0.065) | **0.018** | -0.414 (-0.685, -0.143) | **0.003** | -0.067 (-0.114, -0.019) | **0.007** |
| Neu | Ref. | -0.278 (-0.552, -0.005) | **0.046** | -0.414 (-0.695, -0.133) | **0.004** | -0.054 (-0.094, -0.013) | **0.010** | -0.293 (-0.548, -0.038) | **0.025** | -0.399 (-0.668, -0.129) | **0.004** | -0.057 (-0.100, -0.015) | **0.009** |
| Lym | Ref. | -0.049 (-0.168, 0.070) | 0.413 | -0.032 (-0.165, 0.101) | 0.630 | -0.005 (-0.018, 0.009) | 0.471 | -0.029 (-0.133, 0.076) | 0.583 | 0.011 (-0.117, 0.138) | 0.867 | -0.003 (-0.016, 0.009) | 0.591 |
| Mono | Ref. | -0.028 (-0.062, 0.005) | 0.096 | -0.039 (-0.076, -0.002) | **0.041** | -0.006 (-0.011, -0.001) | **0.011** | -0.033 (-0.064, -0.002) | **0.037** | -0.022 (-0.060, 0.017) | 0.269 | -0.004 (-0.009, 0.001) | 0.145 |
| NLR | Ref. | -0.140 (-0.360, 0.081) | 0.210 | -0.269 (-0.480, -0.058) | **0.013** | -0.037 (-0.061, -0.013) | **0.003** | -0.163 (-0.373, 0.046) | 0.124 | -0.275 (-0.473, -0.077) | **0.007** | -0.035 (-0.059, -0.012) | **0.004** |
| MLR | Ref. | -0.013 (-0.037, 0.011) | 0.291 | -0.031 (-0.053, -0.009) | **0.006** | -0.004 (-0.007, -0.002) | **0.003** | -0.018 (-0.041, 0.006) | 0.135 | -0.026 (-0.047, -0.006) | **0.013** | -0.003 (-0.006, 0.000) | **0.037** |
| PLR | Ref. | -0.473 ( -8.699, 7.754) | 0.909 | -5.615 (-13.600, 2.369) | 0.165 | -1.232 (-2.240, -0.224) | **0.017** | 0.746 ( -7.271, 8.762) | 0.853 | -7.627 (-15.041, -0.212) | **0.044** | -1.458 ( -2.366, -0.550) | **0.002** |
| PIV | Ref. | -42.483 ( -93.432, 8.466) | 0.101 | -76.732 (-127.056, -26.407) | **0.003** | -10.998 (-17.834, -4.162) | **0.002** | -42.616 ( -91.061, 5.828) | 0.084 | -74.675 (-123.447, -25.904) | **0.003** | -10.851 ( -17.876, -3.825) | **0.003** |
| SIRI | Ref. | -0.155 (-0.333, 0.023) | 0.087 | -0.259 (-0.441, -0.077) | **0.006** | -0.035 (-0.060, -0.009) | **0.009** | -0.178 (-0.346, -0.009) | **0.039** | -0.237 (-0.415, -0.060) | **0.010** | -0.03 (-0.057, -0.003) | **0.032** |
| SII | Ref. | -42.700 (-99.970, 14.570) | 0.142 | -89.159 (-146.014, -32.303) | **0.003** | -13.708 (-20.817, -6.598) | **<0.001** | -39.678 ( -94.460, 15.105) | 0.153 | -97.05 (-149.552, -44.548) | **<0.001** | -15.25 ( -21.867, -8.634) | **<0.001** |
| **Biomarkers of lipid** (n = 685 for LDL,  n = 1,520 for others) |  |  |  |  |  |  |  |  |  |  |  |  |  |
| TC | Ref. | -7.193 (-16.474, 2.089) | 0.127 | -4.765 (-14.090, 4.559) | 0.312 | -0.548 (-1.746, 0.650) | 0.365 | -6.367 (-15.542, 2.807) | 0.170 | -2.614 (-11.433, 6.204) | 0.556 | -0.343 ( -1.504, 0.819) | 0.558 |
| TG | Ref. | -21.612 (-45.999, 2.774) | 0.082 | -20.394 (-42.944, 2.156) | 0.076 | -1.953 (-4.939, 1.034) | 0.197 | -22.273 (-43.217, -1.329) | **0.038** | -21.190 (-41.948, -0.432) | **0.046** | -2.539 ( -5.556, 0.478) | 0.098 |
| HDL | Ref. | -2.242 (-7.113, 2.629) | 0.362 | 0.120 (-4.863, 5.102) | 0.962 | -0.132 (-0.839, 0.575) | 0.711 | -1.745 ( -6.479, 2.989) | 0.464 | -0.884 ( -5.595, 3.828) | 0.709 | -0.285 ( -0.967, 0.397) | 0.407 |
| LDL | Ref. | 0.138 (-9.198, 9.474) | 0.977 | 4.303 (-4.331, 12.937) | 0.324 | 0.002 (-0.009, 0.014) | 0.697 | 1.171 ( -7.732, 10.075) | 0.793 | 7.027 (-1.135, 15.190) | 0.090 | 0.006 (-0.005, 0.017) | 0.258 |
| **Biomarkers of renal function** (n = 1,519) |  |  |  |  |  |  |  |  |  |  |  |  |  |
| eGFR | Ref. | 6.838 (2.686, 10.990) | **0.002** | 12.850 (9.294, 6.407) | **<0.001** | 1.81 (1.327, 2.293) | **<0.001** | 5.917 (1.772, 10.062) | **0.006** | 8.476 (5.046, 11.906) | **<0.001** | 1.148 (0.679, 1.617) | **<0.001** |
| Serum urea nitrogen | Ref. | -1.630 (-2.727, -0.533) | **0.004** | -3.071 (-4.138, -2.003) | **<0.001** | -0.42 (-0.569, -0.271) | **<0.001** | -1.586 (-2.562, -0.611) | **0.002** | -2.499 (-3.451, -1.548) | **<0.001** | -0.331 (-0.460, -0.202) | **<0.001** |
| Serum creatinine | Ref. | -0.159 (-0.263, -0.054) | **0.003** | -0.242 (-0.347, -0.138) | **<0.001** | -0.034 (-0.049, -0.020) | **<0.001** | -0.167 (-0.270, -0.065) | **0.002** | -0.231 (-0.343, -0.119) | **<0.001** | -0.030 (-0.046, -0.015) | **<0.001** |
| Uric acid | Ref. | -0.187 (-0.486, 0.112) | 0.217 | -0.739 (-1.014, -0.464) | **<0.001** | -0.099 (-0.137, -0.061) | **<0.001** | -0.245 (-0.507, 0.018) | 0.067 | -0.581 (-0.829, -0.332) | **<0.001** | -0.070 (-0.106, -0.034) | **<0.001** |
| UACR | Ref. | -67.879 (-170.979, 35.221) | 0.194 | -64.025 (-161.076, 33.027) | 0.193 | -0.059 (-0.180, 0.061) | 0.331 | -58.260 (-152.786, 36.266) | 0.223 | -88.478 (-181.002, 4.046) | 0.061 | -0.097 (-0.219, 0.026) | 0.120 |

The unadjusted model was not adjusted by any covariates. The adjusted model was fully adjusted for sex, age, race, educational attainment, BMI, smoking status, CVD, DM and hypertension. CI, confidence interval; T1, the lowest tertile of Klotho level; T2, the moderate tertile of Klotho level; T3, the highest tertile of Klotho level; Ref, reference.
