# Supplementary material for: Correction to: Klotho exerts protection in chronic kidney disease associated with regulating inflammatory response and lipid metabolism
Source: Cell Biosci. 2024 Jul 26;14:97. doi: 10.1186/s13578-024-01263-z (PMC11282863; doi:10.1186/s13578-024-01263-z)
Supplement: Supplementary file 3 — Supplementary Material 3 [file 13578_2024_1263_MOESM3_ESM.docx]

**Table S4** Mediation effects of inflammation and lipid biomarkers on the association of Klotho with renal function in general population.

| **Outcomes** | **Mediators** | **Indirect effect** | **Direct effect** | **Total effect** | **Mediated proportion (%)** | **P-value** |
| --- | --- | --- | --- | --- | --- | --- |
| **Biomarkers of renal function** | **Biomarkers of inflammation** | β (95% CI) | β (95% CI) | β (95% CI) |  |  |
| **eGFR** | WBC | 0.00014 (0.00006, 0.00025) *** | 0.00365 (0.00250, 0.00479) *** | 0.00379 (0.00266, 0.00479) *** | 3.749 | **<0.001** |
|  | Neu | 0.00015 (0.00007, 0.00025) *** | 0.00363 (0.00251, 0.00475) *** | 0.00378 (0.00266, 0.00488) *** | 3.747 | **<0.001** |
|  | Lym | 0.00001 (-0.00001, 0.00005) | 0.00382 (0.00270, 0.00492) *** | 0.00158 (0.00274, 0.00494) *** | 0.158 | 0.516 |
|  | Mono | 0.00002 (-0.00001, 0.00006) | 0.00379 (0.00262, 0.00491) *** | 0.00381 (0.00266, 0.00492) *** | 0.322 | 0.256 |
|  | NLR | 0.00008 (0.00001, 0.00017) * | 0.00370 (0.00248, 0.00484) *** | 0.00378 (0.00257, 0.00496) *** | 2.073 | **0.036** |
|  | MLR | 0.00001 (-0.00001, 0.00004) | 0.00379 (0.00268, 0.00490) *** | 0.00379 (0.00271, 0.00491) *** | 0.134 | 0.556 |
|  | PLR | -0.00005 (-0.00015, 0.00002) | 0.00387 (0.00277, 0.00494) *** | 0.00382 (0.00272, 0.00489) *** | NA | 0.188 |
|  | PIV | 0.00007 (-0.00000, 0.00014) | 0.00371 (0.00254, 0.00479) *** | 0.00377 (0.00260, 0.00487) *** | 1.712 | 0.060 |
|  | SIRI | 0.00006 (0.00000, 0.00014) * | 0.00373 (0.00261, 0.00499) *** | 0.00379 (0.00265, 0.00505) *** | 1.448 | **0.020** |
|  | SII | 0.00010 (0.00002, 0.00019) * | 0.00373 (0.00265, 0.00483) *** | 0.00382 (0.00275, 0.00497) *** | 2.375 | **0.024** |
|  | **Biomarkers of lipid** |  |  |  |  |  |
|  | TC | 0.00001 (-0.00011, 0.00011) | 0.00364 (0.00247, 0.00484) *** | 0.00365 (0.00249, 0.00484) *** | 0.189 | 0.884 |
|  | TG | 0.00006 (-0.00001, 0.00015) | 0.00367 (0.00254, 0.00479) *** | 0.00372 (0.00260, 0.00481) *** | 1.351 | 0.092 |
|  | HDL | -0.00009 (-0.00021, 0.00000) | 0.00382 (0.00263, 0.00493) *** | 0.00374 (0.00253, 0.00482) *** | NA | 0.056 |
|  | LDL | 0.00002 (-0.00005, 0.00011) | 0.00228 (0.00079, 0.00373) *** | 0.00229 (0.00081, 0.00375) *** | 0.400 | 0.636 |
| **Serum urea nitrogen** | **Biomarkers of inflammation** |  |  |  |  |  |
|  | WBC | -0.00003 (-0.00005, -0.00001) ** | -0.00067 (-0.00103, -0.00032) *** | -0.00069 (-0.00107, -0.00034) ** | 3.594 | **0.008** |
|  | Neu | -0.00002 (-0.00004, -0.00000) ** | -0.00067 (-0.00109, -0.00029) ** | -0.00071 (-0.00109, -0.00030) ** | 2.468 | **0.008** |
|  | Lym | -0.00000 (-0.00001, 0.00001) | -0.00068 (-0.00103, -0.00037) *** | -0.00068 (-0.00104, -0.00038) *** | 0.279 | 0.532 |
|  | Mono | -0.00001 (-0.00003, 0.00000) | -0.00068 (-0.00109, -0.00033) *** | -0.00070 (-0.00111, -0.00034) *** | 1.589 | 0.096 |
|  | NLR | -0.00001 (-0.00002, 0.00000) | -0.00069 (-0.00110, -0.00034) *** | -0.00069 (-0.00110, -0.00034) *** | 0.828 | 0.244 |
|  | MLR | -0.00000 (-0.00001, 0.00000) | -0.00070 (-0.00109, -0.00033) *** | -0.00070 (-0.00109, -0.00032) *** | 0.453 | 0.340 |
|  | PLR | 0.00004 (0.00001, 0.00007) *** | -0.00072 (-0.00111, -0.00033) *** | -0.00069 (-0.00109, -0.00030) *** | NA | **<0.001** |
|  | PIV | -0.00001 (-0.00003, 0.00001) | -0.00068 (-0.00104, -0.00032) *** | -0.00069 (-0.00106, -0.00033) *** | 1.407 | 0.244 |
|  | SIRI | -0.00001 (-0.00003, 0.00000) | -0.00068 (-0.00106, -0.00034) *** | -0.00069 (-0.00107, -0.00035) *** | 1.266 | 0.084 |
|  | SII | -0.00000 (-0.00003, 0.00003) | -0.00070 (-0.00110, -0.00034) *** | -0.00070 (-0.00110, -0.00034) *** | 0.176 | 0.888 |
|  | **Biomarkers of lipid** |  |  |  |  |  |
|  | TC | -0.00001 (-0.00004, 0.00001) | -0.00067 (-0.00103, -0.00025) *** | -0.00068 (-0.00104, -0.00028) *** | 1.811 | 0.360 |
|  | TG | -0.00002 (-0.00005, -0.00001) * | -0.00065 (-0.00103, -0.00030) *** | -0.00068 (-0.00104, -0.00032) *** | 3.382 | **0.016** |
|  | HDL | 0.00001 (-0.00001, 0.00003) | -0.00069 (-0.00105, -0.00033) *** | -0.00066 (-0.00105, -0.00029) *** | NA | 0.292 |
|  | LDL | -0.00001 (-0.00004, 0.00001) | -0.00070 (-0.00130, -0.00019) ** | -0.00071 (-0.00132, -0.00020) ** | 1.156 | 0.344 |
| **Serum creatinine** | **Biomarkers of inflammation** |  |  |  |  |  |
|  | WBC | -0.00000 (-0.00000, -0.00000) *** | -0.00007 (-0.00009, -0.00005) *** | -0.00007 (-0.00009, -0.00005) *** | 2.482 | **<0.001** |
|  | Neu | -0.00000 (-0.00000, -0.00000) *** | -0.00007 (-0.00009, -0.00005) *** | -0.00007 (-0.00009, -0.00005) *** | 3.158 | **<0.001** |
|  | Lym | 0.00000 (-0.00000, 0.00000) | -0.00007 (-0.00009, -0.00005) *** | -0.00007 (-0.00009, -0.00005) *** | NA | 0.736 |
|  | Mono | -0.00000 (-0.00000, 0.00000) | -0.00007 (-0.00009, -0.00005) *** | -0.00007 (-0.00009, -0.00005) *** | 0.255 | 0.404 |
|  | NLR | -0.00000 (-0.00000, -0.00000) * | -0.00007 (-0.00009, -0.00005) *** | -0.00007 (-0.00009, -0.00005) *** | 2.808 | **0.032** |
|  | MLR | -0.00000 (-0.00000, 0.00000) | -0.00007 (-0.00009, -0.00005) *** | -0.00007 (-0.00009, -0.00005) *** | 0.478 | 0.268 |
|  | PLR | -0.00000 (-0.00000, 0.00000) | -0.00007 (-0.00010, -0.00005) *** | -0.00007 (-0.00010, -0.00005) *** | 0.294 | 0.788 |
|  | PIV | -0.00000 (-0.00000, -0.00000) * | -0.00007 (-0.00009, -0.00005) *** | -0.00007 (-0.00009, -0.00005) *** | 1.913 | **0.020** |
|  | SIRI | -0.00000 (-0.00000, -0.00000) ** | -0.00007 (-0.00009, -0.00005) *** | -0.00007 (-0.0009, -0.00005) *** | 1.801 | **0.004** |
|  | SII | -0.00000 (-0.00000, -0.00000) * | -0.00007 (-0.00009, -0.00005) *** | -0.00007 (-0.00009, -0.00005) *** | 3.113 | **0.016** |
|  | **Biomarkers of lipid** |  |  |  |  |  |
|  | TC | 0.00000 (-0.00000, 0.00000) | -0.00007 (-0.00009, -0.00005) *** | -0.00007 (-0.00009, -0.00005) *** | NA | 0.288 |
|  | TG | -0.00000 (-0.00000, 0.00000) | -0.00007 (-0.00009, -0.00005) *** | -0.00007 (-0.00009, -0.00005) *** | NA | 0.160 |
|  | HDL | 0.00000 (0.00000, 0.00000) *** | -0.00007 (-0.00009, -0.00005) *** | -0.00007 (-0.00009, -0.00004) *** | NA | 0.092 |
|  | LDL | 0.00000 (-0.00000, 0.00000) | -0.00005 (-0.00007, -0.00002) *** | -0.00005 (-0.00007, -0.00002) *** | NA | 0.560 |
| **Uric acid** | **Biomarkers of inflammation** |  |  |  |  |  |
|  | WBC | -0.00001 (-0.00002, -0.00000) ** | -0.00045 (-0.00057, -0.00035) *** | -0.00047 (-0.00058, -0.00036) *** | 2.134 | **0.004** |
|  | Neu | -0.00001 (-0.00002, -0.00000) *** | -0.00045 (-0.00056, -0.00035) *** | -0.00046 (-0.00057, -0.00036) *** | 1.749 | **<0.001** |
|  | Lym | -0.00000 (-0.00001, 0.00000) | -0.00046 (-0.00057, -0.00035) *** | -0.00047 (-0.00057, -0.00035) *** | 0.220 | 0.420 |
|  | Mono | -0.00000 (-0.00001, 0.00000) | -0.00046 (-0.00057, -0.00035) *** | -0.00046 (-0.00057, -0.00036) *** | 0.718 | 0.092 |
|  | NLR | -0.00000 (-0.00001, 0.00000) | -0.00046 (-0.00059, -0.00035) *** | -0.00047 (-0.00059, -0.00035) *** | 0.256 | 0.392 |
|  | MLR | -0.00000 (-0.00000, 0.00000) | -0.00046 (-0.00058, -0.00037) *** | -0.00046 (-0.00058, -0.00037) *** | 0.115 | 0.436 |
|  | PLR | 0.00001 (-0.00000, 0.00002) | -0.00047 (-0.00057, -0.00037) *** | -0.00046 (-0.00057, -0.00036) *** | NA | 0.160 |
|  | PIV | -0.00001 (-0.00001, -0.00000) | -0.00046 (-0.00057, -0.00035) *** | -0.00046 (-0.00058, -0.00036) *** | 1.220 | 0.104 |
|  | SIRI | -0.00000 (-0.00001, -0.00000) * | -0.00046 (-0.00059, -0.00035) *** | -0.00046 (-0.00059, -0.00036) *** | 0.688 | **0.048** |
|  | SII | -0.00000 (-0.00001, -0.00000) | -0.00046 (-0.00057, -0.00035) *** | -0.00047 (-0.00058, -0.00036) *** | 0.810 | 0.248 |
|  | **Biomarkers of lipid** |  |  |  |  |  |
|  | TC | -0.00002 (-0.00003, -0.00001) *** | -0.00043 (-0.00054, -0.00032) *** | -0.00045 (-0.00056, -0.00034) *** | 4.349 | **<0.001** |
|  | TG | -0.00001 (-0.00003, 0.00000) * | -0.00044 (-0.00055, -0.00034) *** | -0.00045 (-0.00057, -0.00035) *** | 2.511 | **0.016** |
|  | HDL | 0.00001 (-0.00000, 0.00002) | -0.00047 (-0.00058, -0.00036) *** | -0.00046 (-0.00057, -0.00035) *** | NA | 0.104 |
|  | LDL | -0.00001 (-0.00002, 0,00000) | -0.00041 (-0.00054, -0.00028) *** | -0.00041 (-0.00055, -0.00028) *** | 1.555 | 0.220 |
| **UACR** | **Biomarkers of inflammation** |  |  |  |  |  |
|  | WBC | -0.00047 (-0.00127, 0.00023) | -0.01460 (-0.03080, 0.00131) | -0.01507 (-0.03155, 0.00143) | 2.880 | 0.204 |
|  | Neu | -0.00071 (-0.00184, 0.00018) | -0.01429 (-0.03077, 0.00158) | -0.01500 (-0.03161, 0.00180) | 4.316 | 0.172 |
|  | Lym | 0.00007 (-0.00016, 0.00047) | -0.01509 (-0.03162, 0.00072) | -0.01502 (-0.03157, 0.00076) | NA | 0.702 |
|  | Mono | -0.00001 (-0.00026, 0.00022) | -0.01535 (-0.03226, 0.00061) | -0.01536 (-0.03215, 0.00073) | 0.003 | 0.922 |
|  | NLR | -0.00056 (-0.00131, -0.00003) * | -0.01482 (-0.03072, -0.00011) * | -0.01538 (-0.03153, -0.00064) * | 3.433 | 0.060 |
|  | MLR | -0.00014 (-0.00060, 0.00020) | -0.01508 (-0.03176, 0.00059) | -0.01522 (-0.03203, 0.00053) | 0.627 | 0.508 |
|  | PLR | -0.00061 (-0.00175, 0.00041) | -0.01412 (-0.03032, 0.00320) | -0.01476 (-0.03110, 0.00296) | 3.567 | 0.328 |
|  | PIV | -0.00040 (-0.00113, 0.00024) | -0.01441 (-0.03024, 0.00119) | -0.01481 (-0.03068, 0.00076) | 2.431 | 0.302 |
|  | SIRI | -0.00039 (-0.00105, 0.00006) | -0.01483 (-0.03125, -0.00002) * | -0.01512 (-0.03169, -0.00049) * | 2.295 | 0.120 |
|  | SII | -0.00071 (-0.00160, 0.00011) | -0.01406 (-0.03069, 0.00336) | -0.01478 (-0.03133, 0.00256) | 4.403 | 0.168 |
|  | **Biomarkers of lipid** |  |  |  |  |  |
|  | TC | -0.00154 (-0.00397, 0.00029) | -0.01357 (-0.03032, 0.00263) | -0.01511 (-0.03170, 0.00093) | 9.295 | 0.150 |
|  | TG | -0.00114 (-0.00312, 0.00025) | -0.01387 (-0.03056, 0.00275) | -0.01501 (-0.03157, 0.00143) | 6.551 | 0.168 |
|  | HDL | -0.00016 (-0.00091, 0.00043) | -0.01547 (-0.03195, 0.00208) | -0.01563 (-0.03212, 0.00187) | 0.623 | 0.650 |
|  | LDL | -0.00011 (-0.00083, 0.00046) | -0.00257 (-0.01944, 0.01509) | -0.00268 (-0.01963, 0.01475) | 0.433 | 0.814 |

The model was fully adjusted for sex, age, race, educational attainment, BMI, smoking status, CVD, DM and hypertension. CI, confidence interval; NA, represents a too small percentage. * p < 0.05, ** p < 0.01 and *** p < 0.001.
