# Supplementary material for: Correction to: Klotho exerts protection in chronic kidney disease associated with regulating inflammatory response and lipid metabolism
Source: Cell Biosci. 2024 Jul 26;14:97. doi: 10.1186/s13578-024-01263-z (PMC11282863; doi:10.1186/s13578-024-01263-z)
Supplement: Supplementary file 4 — Supplementary Material 4 [file 13578_2024_1263_MOESM4_ESM.docx]

**Table S5** Mediation effects of inflammation and lipid biomarkers on the association of Klotho with renal function in CKD population.

| **Outcomes** | **Mediators** | **Indirect effect** | **Direct effect** | **Total effect** | **Mediated proportion (%)** | **P-value** |
| --- | --- | --- | --- | --- | --- | --- |
| **Biomarkers of renal function** | **Biomarkers of inflammation** | β (95% CI) | β (95% CI) | β (95% CI) |  |  |
| **eGFR** | WBC | 0.00044 (0.00006, 0.00090) * | 0.01139 (0.00639, 0.01519) *** | 0.01139 (0.00688, 0.01615) *** | 3.478 | **0.012** |
|  | Neu | 0.00042 (0.00002, 0.00096) * | 0.01114 (0.00677, 0.01581) *** | 0.01156 (0.00706, 0.01624) *** | 3.449 | **0.028** |
|  | Lym | 0.00002 (-0.00005, 0.00013) | 0.01154 (0.00700, 0.01641) *** | 0.01157 (0.00702, 0.01645) *** | 0.136 | 0.592 |
|  | Mono | 0.00009 (-0.00007, 0.00039) | 0.01174 (0.00696, 0.01595) *** | 0.01183 (0.00708, 0.01613) *** | 0.523 | 0.392 |
|  | NLR | 0.00024 (-0.00009, 0.00069) | 0.01151 (0.00706, 0.01609) *** | 0.01175 (0.00741, 0.01621) *** | 1.715 | 0.156 |
|  | MLR | 0.00002 (-0.00023, 0.00028) | 0.01168 (0.00684, 0.01621) *** | 0.01170 (0.00691, 0.01615) *** | 0.063 | 0.920 |
|  | PLR | -0.00010 (-0.00044, 0.0002) | 0.01184 (0.00728, 0.01685) *** | 0.01174 (0.00727, 0.01671) *** | NA | 0.492 |
|  | PIV | 0.00019 (-0.00002, 0.00046) | 0.0145 (0.00687, 0.01614) *** | 0.01163 (0.00713, 0.01629) *** | 1.545 | 0.076 |
|  | SIRI | 0.00021 (-0.00007, 0.00064) | 0.01138 (0.00701, 0.01596) *** | 0.01160 (0.00715, 0.01624) *** | 1.587 | 0.168 |
|  | SII | 0.00021 (-0.00003, 0.00053) | 0.01144 (0.00713, 0.01586) *** | 0.01165 (0.00738, 0.01603) *** | 1.633 | 0.084 |
|  | **Biomarkers of lipid** |  |  |  |  |  |
|  | TC | -0.00007 (-0.00051, 0.00025) | 0.01167 (0.00729, 0.01612) *** | 0.01161 (0.00709, 0.01606) *** | NA | 0.732 |
|  | TG | 0.00012 (-0.00011, 0.00049) | 0.01141 (0.00711, 0.01575) *** | 0.01153 (0.00707, 0.01593) *** | 0.768 | 0.372 |
|  | HDL | -0.00027 (-0.00112, 0.00036) | 0.01192 (0.00729, 0.01652) *** | 0.01164 (0.00707, 0.01620) *** | NA | 0.448 |
|  | LDL | 0.00004 (-0.00038, 0.00049) | 0.00893 (0.00205, 0.01558) * | 0.00898 (0.00225, 0.01555) * | 0.271 | 0.820 |
| **Blood urea nitrogen** | **Biomarkers of inflammation** |  |  |  |  |  |
|  | WBC | -0.00010 (-0.00026, -0.00000) * | -0.00323 (-0.00455, -0.00195) *** | -0.00334 (-0.00465, -0.00209) *** | 2.858 | **0.048** |
|  | Neu | -0.00010 (-0.00029, 0.00000) | -0.00330 (-0.00453, -0.00204) *** | -0.00341 (-0.00464, -0.00211) *** | 2.673 | 0.060 |
|  | Lym | -0.00000 (-0.00003, 0.00002) | -0.00339 (-0.00467, -0.00213) *** | -0.00339 (-0.00467, -0.00213) *** | 0.013 | 0.872 |
|  | Mono | -0.00004 (-0.00018, 0.00003) | -0.00333 (-0.00460, -0.00209) *** | -0.00337 (-0.00463, -0.00213) *** | 1.026 | 0.328 |
|  | NLR | -0.00007 (-0.00019, 0.00001) | -0.00325 (-0.00451, -0.00210) *** | -0.00332 (-0.00456, -0.00215) *** | 1.959 | 0.104 |
|  | MLR | -0.00009 (-0.00022, 0.00000) | -0.00331 (-0.00454, -0.00217) *** | -0.00340 (-0.00460, -0.00223) *** | 2.378 | 0.060 |
|  | PLR | -0.00003 (-0.00015, 0.00006) | -0.00339 (-0.00478, -0.00223) *** | -0.00342 (-0.00479, -0.00223) *** | 0.713 | 0.508 |
|  | PIV | -0.00009 (-0.00022, 0.00001) | -0.00325 (-0.00441, -0.00194) *** | -0.00333 (-0.00447, -0.00207) *** | 2.474 | 0.076 |
|  | SIRI | -0.00011 (-0.00029, 0.00001) | -0.00328 (-0.00463, -0.00207) *** | -0.00339 (-0.00472, -0.00222) *** | 3.008 | 0.068 |
|  | SII | -0.00008 (-0.00018, 0.00002) | -0.00329 (-0.00454, -0.00196) *** | -0.00337 (-0.00464, -0.00204) *** | 2.268 | 0.100 |
|  | **Biomarkers of lipid** |  |  |  |  |  |
|  | TC | -0.00001 (-0.00010, 0.00007) | -0.00333 (-0.00468, -0.00211) *** | -0.00334 (-0.00466, -0.00213) *** | 0.017 | 0.928 |
|  | TG | -0.00001 (-0.00011, 0.00006) | -0.00334 (-0.00448, -0.00219) *** | -0.00335 (-0.00449, -0.00220) *** | 0.117 | 0.848 |
|  | HDL | 0.00002 (-0.00009, 0.00018) | 0.00331 (-0.00451, -0.00209) *** | -0.00329 (-0.00447, -0.00208) *** | NA | 0.688 |
|  | LDL | 0.00003 (-0.00009, 0.00022) | -0.00324 (-0.00515, -0.00134) ** | -0.00321 (-0.00510, -0.00134) ** | NA | 0.672 |
| **Serum creatinine** | **Biomarkers of inflammation** |  |  |  |  |  |
|  | WBC | -0.00000 (-0.00001, 0.00000) | -0.00030 (-0.00045, -0.00015) *** | -0.00030 (-0.00046, -0.00016) *** | 1.172 | 0.336 |
|  | Neu | -0.00001 (-0.00002, 0.00000) | -0.00030 (-0.00044, -0.00016) *** | -0.00031 (-0.00044, -0.00017) *** | 2.176 | 0.076 |
|  | Lym | -0.00000 (-0.00001, 0.00000) | -0.00031 (-0.00046, -0.00016) *** | -0.00031 (-0.00046, -0.00016) *** | 0.128 | 0.760 |
|  | Mono | 0.00000 (-0.00001, 0.00000) | -0.00029 (-0.00041, -0.00019) *** | -0.00031 (-0.00041, -0.00019) *** | 0.111 | 0.680 |
|  | NLR | -0.00002 (-0.00004, 0.00000) | -0.00029 (-0.00044, -0.00014) *** | -0.00031 (-0.00045, -0.00016) *** | 4.524 | 0.088 |
|  | MLR | -0.00001 (-0.00002, -0.00000) * | -0.00029 (-0.00045, -0.00016) *** | -0.00030 (-0.00046, -0.00016) *** | 2.425 | **0.032** |
|  | PLR | -0.00000 (-0.00002, 0.00001) | -0.00031 (-0.00046, -0.00015) *** | -0.00031 (-0.00046, -0.00016) *** | 0.808 | 0.564 |
|  | PIV | -0.00000 (-0.00001, 0.00000) | -0.00030 (-0.00045, -0.00014) *** | -0.00031 (-0.00046, -0.00015) *** | 0.766 | 0.340 |
|  | SIRI | -0.00001 (-0.00002, -0.00000) * | -0.00030 (-0.00046, -0.00015) *** | -0.00031 (-0.00047, -0.00016) *** | 2.447 | **0.024** |
|  | SII | -0.00001 (-0.00002, 0.00000) | -0.00030 (-0.00046, -0.00015) *** | -0.00031 (-0.00046, -0.00015) *** | 1.983 | 0.248 |
|  | **Biomarkers of lipid** |  |  |  |  |  |
|  | TC | 0.00000 (-0.00001, 0.00002) | -0.00031 (-0.00046, -0.00014) *** | -0.00031 (-0.00046, -0.00014) *** | NA | 0.728 |
|  | TG | -0.00000 (-0.00001, 0.00001) | -0.00031 (-0.00047, -0.00015) *** | -0.00031 (-0.00047, -0.00015) *** | 0.029 | 0.932 |
|  | HDL | 0.00001 (-0.00001, 0.00003) | -0.00031 (-0.00047, -0.00018) *** | -0.00030 (-0.00046, -0.00017) *** | NA | 0.308 |
|  | LDL | -0.00001 (-0.00002, 0.00001) | -0.00023 (-0.00040, -0.00005) * | -0.00024 (-0.00041, -0.00006) ** | 2.344 | 0.308 |
| **Uric acid** | **Biomarkers of inflammation** |  |  |  |  |  |
|  | WBC | -0.00002 (-0.00005, 0.00001) | -0.00069 (-0.00104, -0.00036) *** | -0.00070 (-0.00105, -0.00037) *** | 1.996 | 0.256 |
|  | Neu | -0.00002 (-0.00006, 0.00001) | -0.00067 (-0.00102, -0.00032) *** | -0.00069 (-0.00106, -0.00033) *** | 2.396 | 0.208 |
|  | Lym | 0.00000 (-0.00000, 0.00001) | -0.00069 (-0.00108, -0.00035) *** | -0.00069 (-0.00108, -0.00035) *** | NA | 0.912 |
|  | Mono | -0.00001 (-0.00004, 0.00001) | -0.00070 (-0.00106, -0.00036) *** | -0.00071 (-0.00109, -0.00037) *** | 0.847 | 0.420 |
|  | NLR | -0.00002 (-0.00005, 0.00000) | -0.00069 (-0.00102, -0.00033) *** | -0.00070 (-0.00104, -0.00035) *** | 2.232 | 0.116 |
|  | MLR | -0.00002 (-0.00007, -0.00000) * | -0.00069 (-0.00102, -0.00036) *** | -0.00071 (-0.00106, -0.00038) *** | 3.181 | **0.048** |
|  | PLR | -0.00001 (-0.00004, 0.00002) | -0.00069 (-0.00101, -0.00037) *** | -0.00070 (-0.00101, -0.00039) *** | 1.091 | 0.492 |
|  | PIV | -0.00002 (-0.00005, -0.00001) ** | -0.00066 (-0.00106, -0.00028) *** | -0.00069 (-0.00108, -0.00029) *** | 3.396 | **0.004** |
|  | SIRI | -0.00003 (-0.00008, 0.00000) | -0.00067 (-0.00101, -0.00034) *** | -0.00070 (-0.00104, -0.00036) *** | 3.853 | 0.064 |
|  | SII | -0.00002 (-0.00005, -0.00001) ** | -0.00067 (-0.00102, -0.00033) ** | -0.00069 (-0.00104, -0.00035) *** | 3.412 | **0.008** |
|  | **Biomarkers of lipid** |  |  |  |  |  |
|  | TC | -0.00001 (-0.00005, 0.00003) | -0.00069 (-0.00104, -0.00036) *** | -0.00070 (-0.00106, -0.00038) *** | 1.429 | 0.576 |
|  | TG | -0.00000 (-0.00002, 0.00002) | -0.00068 (-0.00100, -0.00034) *** | -0.00068 (-0.00101, -0.00033) *** | 0.010 | 0.860 |
|  | HDL | 0.00001 (-0.00002, 0.00006) | -0.00070 (-0.00103, -0.00032) *** | -0.00069 (-0.00103, -0.00031) *** | NA | 0.604 |
|  | LDL | 0.00002 (-0.00001, 0.00006) | -0.00059 (-0.00110, -0.00015) ** | -0.00057 (-0.00099, -0.00015) ** | NA | 0.288 |
| **UACR** | **Biomarkers of inflammation** |  |  |  |  |  |
|  | WBC | -0.00164 (-0.00912, 0.00502) | -0.09732 (-0.21733, 0.02440) | -0.09896 (-0.21960, 0.02468) | 1.378 | 0.588 |
|  | Neu | -0.00234 (-0.01276, 0.00707) | -0.09632 (-0.21162, 0.01729) | -0.09865 (-0.21549, 0.01935) | 2.125 | 0.564 |
|  | Lym | 0.00022 (-0.00152, 0.00255) | -0.09537 (-0.20793, 0.02338) | -0.09515 (-0.20811, 0.02382) | NA | 0.828 |
|  | Mono | 0.00029 (-0.00259, 0.00334) | -0.09648 (-0.20797, 0.02919) | -0.09619 (-0.20758, 0.02941) | NA | 0.844 |
|  | NLR | -0.00302 (-0.01200, 0.00387) | -0.09315 (-0.21603, 0.02642) | -0.09617 (-0.21872, 0.02457) | 2.348 | 0.484 |
|  | MLR | -0.00357 (-0.01416, 0.00452) | -0.09408 (-0.20872, 0.01801) | -0.09765 (-0.21176, 0.01138) | 2.642 | 0.424 |
|  | PLR | -0.00240 (-0.01137, 0.00506) | -0.09423 (-0.20842, 0.03171) | -0.09663 (-0.21139, 0.02451) | 1.685 | 0.612 |
|  | PIV | 0.00036 (-0.00423, 0.00533) | -0.09879 (-0.21538, 0.02442) | -0.09843 (-0.21467, 0.02326) | NA | 0.832 |
|  | SIRI | -0.00208 (-0.01315, 0.00576) | -0.09804 (-0.22500, 0.02109) | -0.10012 (-0.22824, 0.02339) | 1.085 | 0.660 |
|  | SII | -0.00011 (-0.00538, 0.00474) | -0.09830 (-0.22192 0.02074) | -0.09841 (-0.21721, 0.01953) | 0.096 | 0.960 |
|  | **Biomarkers of lipid** |  |  |  |  |  |
|  | TC | -0.00361 (-0.01906, 0.00734) | -0.09290 (-0.20930, 0.02344) | -0.09652 (-0.21285, 0.01829) | 2.214 | 0.614 |
|  | TG | -0.00677 (-0.02531, 0.00511) | -0.09180 (-0.20523, 0.02154) | -0.09857 (-0.21299, 0.01691) | 5.276 | 0.342 |
|  | HDL | -0.00134 (-0.01205, 0.00711) | -0.09625 (-0.21968, 0.02842) | -0.09759 (-0.21909, 0.02652) | 0.278 | 0.854 |
|  | LDL | 0.00078 (-0.00487, 0.00799) | -0.02487 (-0.15425, 0.10331) | -0.02408 (-0.15220, 0.10381) | NA | 0.974 |

The model was fully adjusted for sex, age, race, educational attainment, BMI, smoking status, CVD, DM and hypertension. CI, confidence interval; NA, represents a too small percentage. * p < 0.05, ** p < 0.01 and *** p < 0.001.
